# Is cell segregation like oil and water: Asymptotic versus transitory regime

**DOI:** 10.1371/journal.pcbi.1010460

**Published:** 2022-09-19

**Authors:** Florian Franke, Sebastian Aland, Hans-Joachim Böhme, Anja Voss-Böhme, Steffen Lange

**Affiliations:** 1 DataMedAssist, HTW Dresden, Dresden, Germany; 2 Faculty of Informatics/Mathematics, HTW Dresden - University of Applied Sciences, Dresden, Germany; 3 Faculty of Mathematics and Computer Science, TU Freiberg, Freiberg, Germany; Leiden University Faculty of Science: Universiteit Leiden Faculteit der Wiskunde en Natuurwetenschappen, NETHERLANDS

## Abstract

Understanding the segregation of cells is crucial to answer questions about tissue formation in embryos or tumor progression. Steinberg proposed that separation of cells can be compared to the separation of two liquids. Such a separation is well described by the Cahn-Hilliard (CH) equations and the segregation indices exhibit an algebraic decay with exponent 1/3 with respect to time. Similar exponents are also observed in cell-based models. However, the scaling behavior in these numerical models is usually only examined in the asymptotic regime and these models have not been directly applied to actual cell segregation data. In contrast, experimental data also reveals other scaling exponents and even slow logarithmic scaling laws. These discrepancies are commonly attributed to the effects of collective motion or velocity-dependent interactions. By calibrating a 2D cellular automaton (CA) model which efficiently implements a dynamic variant of the differential adhesion hypothesis to 2D experimental data from Méhes et al., we reproduce the biological cell segregation experiments with just adhesive forces. The segregation in the cellular automaton model follows a logarithmic scaling initially, which is in contrast to the proposed algebraic scaling with exponent 1/3. However, within the less than two orders of magnitudes in time which are observable in the experiments, a logarithmic scaling may appear as a pseudo-algebraic scaling. In particular, we demonstrate that the cellular automaton model can exhibit a range of exponents ≤1/3 for such a pseudo-algebraic scaling. Moreover, the time span of the experiment falls into the transitory regime of the cellular automaton rather than the asymptotic one. We additionally develop a method for the calibration of the 2D Cahn-Hilliard model and find a match with experimental data within the transitory regime of the Cahn-Hilliard model with exponent 1/4. On the one hand this demonstrates that the transitory behavior is relevant for the experiment rather than the asymptotic one. On the other hand this corroborates the ambiguity of the scaling behavior, when segregation processes can be only observed on short time spans.

## Author summary

Segregation of different cell types is a crucial process for the pattern formation in tissues, in particular during embryogenesis. Since the involved cell interactions are complex and difficult to measure individually in experiments, mathematical modelling plays an increasingly important role to unravel the mechanisms governing segregation. The analysis of these theoretical models focuses mainly on the asymptotic behavior at large times, in a steady regime and for large numbers of cells. Most famously, cell-segregation models based on the minimization of the total surface energy, a mechanism also driving the demixing of immiscible fluids, are known to exhibit asymptotically a particular algebraic scaling behavior. However, it is not clear, whether the asymptotic regime of the numerical models is relevant at the spatio-temporal scales of actual biological processes and in-vitro experiments. By developing a mapping between 2D cell-based models and experimental settings, we are able to directly compare previous experimental data to numerical simulations of cell segregation quantitatively. We demonstrate that the experiments are reproduced by the transitory regime of the models rather than the asymptotic one. Our work puts a new perspective on previous model-driven conclusions on cell segregation mechanisms.

## Introduction

Pattern formation of cells and cell segregation are complex and crucial processes, in particular in the context of embryogenesis. When different types of cells are intermixed, they start to segregate into homogeneous domains [1–5]. This behavior has been shown for many different cell types in several species, for instance hydra [6, 7], zebra fish [8] and chicken [9, 10]. Why and how cells rearrange themself in a certain way is still not fully understood, and various theories and hypotheses have been formulated to explain the process of cell segregation [5, 8, 11–21].

One of the most well-known theories in the context of cell segregation is the differential adhesion hypothesis of Steinberg [14, 22], which focuses on the impact of adhesion on cell segregation. He proposed that the sorting behavior of cells results from differences in the adhesion strengths between different cell types, which implies that sorting is driven by the minimization of the surface energy. Additionally, he suggested that a mixed cell population will always minimize its total adhesive free energy and conjectured that cells segregate like demixable fluids, e.g., water and oil. Note, that this hypothesis is still debated and alternative, partly related hypotheses where formulated like the differential surface contraction hypothesis [21].

The separation of fluids is theoretically well studied. The kinetics of this separation can be modeled with the Cahn-Hilliard Navier-Stokes equations [23–25]. The level of segregation is typically quantified by segregation indices, the interface length between clusters of different type or by the average cluster diameter. For a narrow cluster size distribution, the average cluster diameter scales inverse-proportional to the interface length and segregation indices, see in S1 Text. An increase of the level of segregation corresponds to a decrease of the former two measures and, accordingly, an increase of the latter one, the average cluster diameter. For the Cahn-Hilliard Navier-Stokes model, it is well known that during segregation the interface length exhibits an algebraic decay over several orders of magnitude in time. The exponent of this algebraic scaling depends on the flows in the model, which is influenced, among others, by the length scale of the system [26], ranging from 1/3 [26–28] for the diffusive regime described by the Lifshitz-Slyozov-Wagner (LSW) theory, into which the scenario of segregating biological cells falls, to 2/3 for the laminar or turbulent regime [26]. Note that, on the temporal scale, these exponents are only reached asymptotically and can be preceded by exponents down to 1/6 in an intermittent regime [28]. In either case, the average cluster diameter is inverse-proportional to the interface length, that is both the cluster diameter and the interface length scale algebraically with exponents that are equal in absolute value but have opposite signs.

In contrast to fluid segregation, not only one but a variety of agent-based models have been used to simulate the segregation of biological cells [15, 29, 30], since there is a variety of cell-based mechanisms, such as active cells or cell interaction mechanisms beside adhesion, which have potential influence on the segregation and need to be studied. While algebraic scalings of the segregation indices over time can be observed in most of these models, the corresponding exponents vary over a wide range of 1/40 − 1/3, see overview Tab A in S1 Text, depending on which segregation mechanisms are incorporated and which models are used [16–18, 31–33]. One of the earliest attempts of simulating cell segregation is the Cellular-Potts-Model (CPM) of Glazier and Graner [12, 13], in which segregation results from differential adhesion. While the observed segregation indices display a logarithmic decay, successive studies concluded that the segregation indices actually follow a logarithmic decay only initially and settle to an algebraic one for longer times [16–18, 32, 34, 35]. Nakajima and Ishihara [17] used the CPM to study the effects of even and uneven cell type ratios on the segregation process. They found the exponent of the algebraic scaling to decrease for increasingly asymmetric mixtures of cells, with exponents ranging from 1/3 for a 50/50 ratio down to 1/4 for a 90/10 ratio. In any case, they observed the average cluster diameter to be inverse-proportional to the segregation indices. Belmonte et al. [16] modeled segregation by a self-propelled particle model with velocity alignment to study the influence of collective motion. They also observed algebraic scaling with an exponent of maximal 0.18 concluding that even weak collective motion accelerates cell segregation. Beatrici et al. [34] used an active particle approach to compare the segregation behavior under different cellular interaction mechanisms including that of the DAH but comprising also related principles with and without collective motion. They measured the average cluster size, which showed an algebraic decay with exponents ranging from 1/2, without collective motion, to 1, with strong collective motion. The latter corresponds to exponents between 1/4 and 1/2 for the average cluster diameter. Beatrici and Brunnet [18] studied a specific particle system incorporating velocity differences between cell types, the boids model, and concluded that velocity differences are sufficient to generate algebraic segregation even without collective motion. Depending on the chosen velocities and cell ratios between fast and slow cell types, they observed both logarithmic and algebraic scaling, the latter with exponents around 1/5, ranging from 0.18 to 0.22. The latter finding is supported by a study of Strandkvist et al. [31] who found an algebraic scaling with exponents ranging from 0.025 to 0.17 with a particle system incorporating velocity differences between cell types. Krajnc [35] used a vertex model to demonstrate that differential fluctuations can efficiently sort cells. He measured the segregation indices over time, which showed a maximal algebraic decay with exponent of 1/4. Durand [32] used a CPM with modified update algorithm, which allows for simulation of larger number of cells over longer times while preserving cell connectivity. He observed an asymptotic algebraic decay with an exponent of 1/4 and concluded that the previously reported scaling with exponent 1/3 is only transitory. He further found the asymptotic scaling to be independent of cell type ratio and boundary conditions.

Concerning data, several experiments have been conducted on cell segregation. Rieu and Sawada [6], Schötz et al. [36] and Beysens et al. [37] conducted experiments with hydra cells and zebra fish cells. They noticed similarities of cell behavior to fluids by comparing characteristics of cell segregation with those expected for viscous fluids according to hydrodynamic laws. For instance, they compared the ratio of viscosity to surface tension and the time course of relaxation to the equilibrium and the characteristics of the reached equilibria. Krieg et al [8] used gastrulating zebrafish embryos cells to quantify adhesive and mechanical properties. While doing so, they also measured the average cluster size over time, which exhibits an algebraic scaling with exponent ∼1/5, corresponding to an exponent 1/10 for the average cluster diameter. Cochet-Escartin et al. [38] studied hydra cells in 3D tissue both in experiments and in CPM simulation to determine whether differences in tissue surface tension are sufficient for segregation. They found algebraic scaling with exponent 0.74 for the experiments and 0.5 for the simulations. However, they only measured cell segregation in the experiments over half an order of magnitude in time. In contrast, Méhes et al. [20] studied the influence of collective motion in experiments with fish and human cells and measured algebraic cell segregation indices with exponent of 0.31 for less than two orders of magnitude in time. They further measured the average cluster diameter, with an algebraic increase with exponents between 0.5 and 0.74. This means that the cluster diameter was not inverse-proportional to the segregation indices, indicating that the cluster size distribution is not narrow. They suspected that this behavior was a result of collective motion, which they concluded to be a segregation promoting effect.

In summary, in the context of cell segregation, an algebraic scaling with an exponent that differs from 1/3, the value expected for fluid segregation, has been attributed to additional intercellular interaction besides differential adhesion [15, 39]. Such mechanisms include collective motion [20, 34, 40] or velocity-dependent interaction of the cells [16, 18, 31, 33]. The analysis of the asymptotic behavior in these theoretical models, in a steady regime and for large numbers of cells, is primarily used to discriminate between models. However, it is unclear whether this asymptotic regime is relevant for biological cell segregation processes and the corresponding in-vitro experiments [32]. Moreover, for both experiments and numerical simulations, the algebraic decay of the segregation indices is usually only observed during the last two orders of magnitude of time [17, 18, 31, 33] or on an even shorter time interval [20, 38].

**Fig 1:**
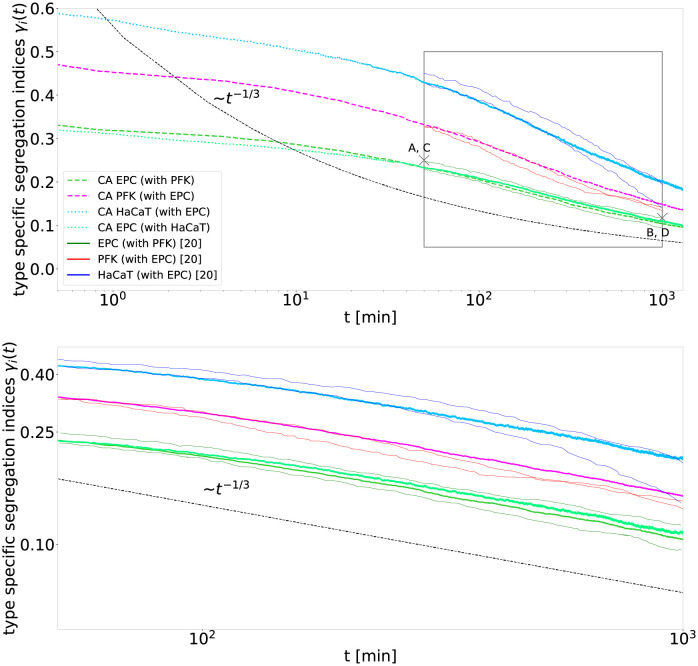
The cellular automaton simulations reproduce the biological cell segregation experiments of Méhes et al. [20]. The segregation indices *γ*_*i*_(*t*) for the two experiments PFK (dark red) with EPC (dark green) and HaCaT (dark blue) with EPC, within the observed time interval 50–1000 min, match with the segregation indices predicted by the cellular automaton (lines with corresponding brighter colors, dashed lines for PFK with EPC and dotted lines for HaCaT with EPC). Within the given time interval (grey box in top panel displayed again in bottom panel), the segregation indices seem to decay algebraically with exponent 1/3 (black dashed line) as expected asymptotically for fluid segregation. For the simulation of the segregation indices *γ*_*i*_(*t*) of PFK (*i* = PFK) mixed with EPC (*i* = EPC), we obtain a cell type ratio of *N*_PFK_/*N*_EPC_ = 41.2/58.8 and fit the adhesion parameters (*β*_PFK-PFK_, *β*_EPC-PFK_, *β*_EPC-EPC_) = (−8.06, −6.56, −0.06) and the time scale of migration *τ*_PFK-EPC_ ≈ 4.2 min. For the simulation of the segregation indices *γ*_*i*_(*t*) of HaCaT (*i* = HaCaT) mixed with EPC (*i* = EPC) we obtain a cell type ratio of *N*_HaCaT_/*N*_EPC_ = 35.2/64.8 and fit the parameters (*β*_HaCaT-HaCaT_, *β*_EPC-HaCaT_, *β*_EPC-EPC_) = (−7.93, −5.44, 0.06) and *τ*_EPC-HaCaT_ ≈ 35.1 min. In both cases 140^2^ cells are simulated, comparable to the cells visible in the experiments, starting from a random mixture. Snapshots of the cell mixtures at the points marked with crosses labeled A, C and B, D are displayed in Fig 3.

**Fig 2:**
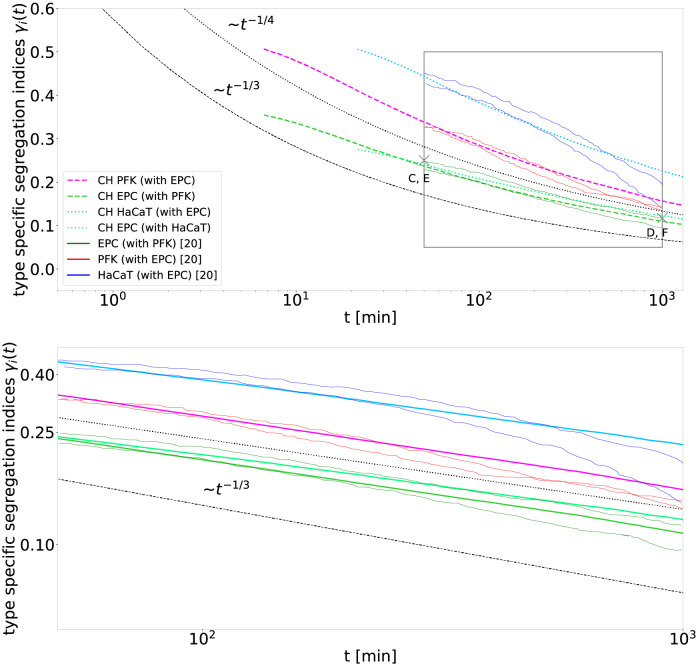
The Cahn-Hilliard simulations reproduce the biological cell segregation experiments of Méhes et al. [20]. The segregation indices *γ*_*i*_(*t*) for the two experiments PFK (dark red) with EPC (dark green) and HaCaT (dark blue) with EPC within the observed time interval 50–1000 min match the segregation indices predicted by the Cahn-Hilliard simulation (lines with corresponding brighter colors, dashed lines for PFK with EPC and dotted lines for HaCaT with EPC). Within the given time interval (grey box in top panel displayed again in bottom panel), the segregation indices of the Cahn-Hilliard simulation decay algebraically with exponent 1/4 (black dotted line) rather than 1/3 (black dashed line), which implies that the segregation process is in an intermittent regime of fluid segregation, see text for details. By using a mapping from the cellular automaton model to the Cahn-Hilliard model, see Materials and methods, parameters are set analogous to the parameters used in Fig 1 except for the mobility constant *D*, which is fitted to $D=36\mu {\text{m}}^{2}/\text{min}$ for the mixture of PFK with EPC and $D=18\mu {\text{m}}^{2}/\text{min}$ for the mixture of HaCaT with EPC. Snapshots of the cell mixtures at the points marked with crosses labeled C, E and D, F are displayed in Fig 3. Note, that the Cahn-Hilliard model is shown after the settling process took place, see Fig E in S1 Text.

We use an efficient implementation of a 2D cellular automaton (CA) model according to Voss-Böhme and Deutsch [19], which solely incorporates adhesive forces between cells, and develop a direct mapping between the model parameters and the experimental setup to reproduce 2D cell segregation experiments from Méhes et al. [20]. We find a match between experimental data and simulations over the whole time span of the experiments, see Fig 1. This is surprising, since our model initially generates logarithmic scaling of the segregation indices over time, see also Fig A in S1 Text. The match between the model and the proposed algebraic scaling with exponent 1/3 in the experiments is possible since the experimental observation is limited to less than two orders of magnitude in time. To make this point more pronounced we will use the term pseudo-algebraic scaling for such behavior in the following. Depending on the model parameters and the considered time interval, we observe this pseudo-algebraic scaling with a range of exponents ≤ 1/3. In the light of such possible misinterpretations, experimental segregation may actually be explained solely by adhesive forces between cells. Thus, we propose that, while additional effects like collective motion might be promoting segregation, the main factor that governs cell segregation may still be adhesive forces. Moreover, we also find a match between the experimental data and the 2D Cahn-Hilliard model, see Fig 2. For this comparison, we develop a mapping between the length scales of the cellular automaton and the Cahn-Hilliard model, such that only a single parameter of the Cahn-Hilliard model, the mobility constant which sets the time scale, has to be fitted. It turns out that the relevant observation window of the experiments falls in the transitory regime of the Cahn-Hilliard model, exhibiting an algebraic scaling exponent of 1/4. Although Méhes et al. [20] suggested an algebraic scaling exponent of 1/3 for the experimental data, we find a good agreement with the Cahn-Hilliard model as well, due to the short observation span. The fact that both models, the cellular automaton and the Cahn-Hilliard model, both agree with the experimental data, while exhibiting different scalings for the experimental setup, corroborates the ambiguity of scaling behavior, when segregation processes are only observed on short time spans. Even more important, the direct application to the experimental setup revealed for both models that the transitory regime of these models is more relevant for the experimental spatio-temporal scales than the asymptotic regime. Since biological experiments are by design restricted to finite time spans, this highlights the importance of considering additional features of segregation beyond the scaling behavior of segregation indices, when comparing with theoretical models.

## Results

### Cellular automaton can reproduce in vitro experiments

We compare our cellular automaton simulations with in-vitro data of Méhes et al. [20], see Fig 1. They measured the segregation indices, cluster sizes, and cluster diameters in the segregation of EPC (fish keratocyte cell line) with PFK (primary goldfish keratocytes) and HaCaT (human keratocyte cell line) with EPC over 1.5 orders of magnitude in time. The cellular automaton has five parameters, which are calibrated to the experimental data: Three adhesion parameters ***β*** = (*β*_00_, *β*_10_, *β*_11_)^*T*^, which set the homotypic (*β*_11_, *β*_00_) and the heterotypic (*β*_01_) adhesion strengths, the cell type ratio *N*_0_/*N*_1_, which reflects the ratio of all numbers *N*_*i*_ of each cell type in the segregation experiments, *i* ∈ {0, 1}, and the time scale of migration *τ*, which relates to the dimensionless time of the cellular automaton to physical time. While *τ* is just a scaling factor for the time, the segregation indices, that should match between the cellular automaton and the experiments, are fixed in their ranges and can not be rescaled. We choose a random initial configuration, which is reasonable with regards to the experiments which also start with mixed cell configurations, while the observations commence a bit later. Note that the three adhesion parameters can be reduced to two effective parameters, the difference of homotypic adhesion *db* and the difference between average homotypic and heterotypic adhesion β*, see Materials and methods for details. In the experiments, equal areas are covered by each cell type, which results in different cell numbers due to slightly different cell sizes for each type. We show that the ratio of cell type numbers *N*_0_/*N*_1_ is set by the ratio of the segregation indices *γ*_1_(*t*)/*γ*_0_(*t*) and thus can be obtained directly from the experimental data, see Eq (13). We check that this ratio is consistent with the ratio of cell sizes of each type and that the total numbers of cells of in the experiments and the simulations are comparable, see Materials and methods.

Simulations and experiments match well for both cell mixtures, see Fig 1. This match is surprising, as the cellular automaton displays in the time frame of the experiments rather a logarithmic scaling, resembling a straight line in the semi-log plot, which contradicts the proposed algebraic scaling of the data in Méhes et al. [20]. However, over just 1.5 orders of magnitude in time a logarithmic decay may appear as almost straight line in a log-log plot as displayed in the bottom panel of Fig 1, making it difficult to distinguish it from a power law. Therefore, we denote an increase or decay which approximately follows a straight line in a log-log plot, but only for a limited time span, as pseudo-algebraic scaling. The match between the prediction of our model and the experiment in Fig 1 demonstrates, that it is not possible to decide in the limited observation time of experiments whether the segregation indices decay algebraically or logarithmically.

For an infinite grid, the asymptotic scaling exponent can only be derived by theoretical arguments [26–28, 41]. Since both, our model system and the experimental system are of finite size, they ultimately settle at a lower bound of the segregation index, which is dependent on the system size. However, we choose a sufficiently large system size for the model, matching that of the experimental setup, such that the lower bound of the segregation indices is at least one to two orders of magnitude smaller than the experimentally observed segregation indices. By this, we avoid finite-size effects and ensure that we can observe the behavior of the segregation indices in the model even after the observation window of the experiments, such assessing whether the scaling still changes. In general, it is elaborate to demonstrate that the numerical behavior of a model is asymptotic. However, for our purpose, it is sufficient to check whether the scaling changes during or after the observation time to determine whether it is still transitory. We denote the last measurable scaling in each simulation as the numerically asymptotic one of the corresponding model, which can still differ from the theoretically expected value for an infinite-size system.

We observe that for the chosen *γ*-fitted parameters the cellular automaton model reaches its asymptotic regime only below the segregation indices *γ* ≈ 0.15, exhibiting an algebraic decay with exponent 1/3 at smaller segregation indices, see Fig A in S1 Text for longer simulations. In contrast, the segregation indices observed in the experiment are higher ranging from 0.5 to 0.1. Therefore, the in-vitro segregation processes fall into the transitory regime of the simulations. In this transitory regime, the model, which uses only adhesive forces, reproduces in-vitro cell segregation. Thus, to explain the observed scaling behavior, it is not necessary to invoke additional complex processes or forces which segregate cells, like collective motion. Note that our main point is to show that the segregation process is not yet in the asymptotic regime, for which it is sufficient to demonstrate that the scaling changes during of after the time period where the segregation indices of the experiment are observed. Eventually, it remains open whether the last scaling observed numerically in the simulation is actually the theoretical asymptotic scaling.

In the model, there is a degree of freedom between the time scale *τ* and the adhesion parameters *β*_*ij*_, see Eq (7). We choose the time scale consistent with the range of reported average velocities of the cells at low density, which are *v*_PFK_ = 500 *μ*m/h, *v*_EPC_ = 30 *μ*m/h, and *v*_HaCaT_ = 34 *μ*m/h [20], such that *τ*_PFK-EPC_ = 2Δ*x*/(*v*_PFK_ + *v*_EPC_) ≈ 4.2 min and *τ*_HaCaT-EPC_ = 2Δ*x*/(*v*_HaCaT_ + *v*_EPC_) ≈ 35.1 min with the average length of a cell $\Delta x\approx \sqrt{350}\mu \text{m}$, see Materials and methods. With this choice, the corresponding adhesion parameters are (*β*_PFK-PFK_, *β*_EPC-PFK_, *β*_EPC-EPC_) = (−8.06, −6.56, −0.06) and (*β*_HaCaT-HaCaT_, *β*_EPC-HaCaT_, *β*_EPC-EPC_) = (−7.93, −5.44, 0.06). Remarkably, we obtain for both experiments, which we fitted independently, similar homotypic adhesion parameters for EPC. While the fitted adhesion parameters may suggest that the homotypic adhesion of HaCaT and PFK is weaker than that of EPC as well as that the homotypic adhesion of HaCaT is equal to that of PFK, this fit is not unique. In fact, due to the short time span the fit is based on, a wide range of adhesion parameters can reproduce the experimental observations, as for instance see parameter variations below. In order to refine the fit, additional data would have to be incorporated, for instance single cell measurements of adhesion forces of each cell type.

### Cahn-Hilliard can reproduce in vitro experiments too

We also compare the segregation experiments of Méhes et al. [20] with fluid segregation. For this, we use the 2D Cahn-Hilliard model which well describes fluid segregation in the diffusive regime in terms of a phase-field formulation, see S1 Text for details. To fit the parameters of the spatially continuous Cahn-Hilliard model to the experimental data, which is based on discrete cells, we develop a mapping between the agent-based cellular automaton and the Cahn-Hilliard model, see S1 Text. Due to this mapping, only the mobility constant *D* of the Cahn-Hilliard model has to be fitted to match the time scale of the experiments, while the remaining parameters can be inferred from the parameters of the cellular automaton used for Fig 1.

2D Cahn-Hilliard simulations and experiments match well for both cell mixtures, see Fig 2. The model fits PFK and EPC very well. However, a small discrepancy can be observed at the end of the fit of HaCaT from the Cahn-Hilliard model, which nevertheless reproduces the data as well as the by Méhes et al. [20] suggested 1/3 algebraic scaling exponent. This match is surprising, as the Cahn-Hilliard simulations rather display an algebraic decay with exponent of 1/4 than 1/3, which was proposed for the data in Méhes et al. [20]. However, within just 1.5 orders of magnitude in time it is hard to distinguish a power-law decay with exponent 1/4 and one with exponent 1/3.

Note that the segregation indices resulting from the Cahn-Hilliard model follow only asymptotically (*t* → ∞) an algebraic scaling with the exponent of 1/3. This asymptotic decay is usually referred to when cell segregation is compared to fluid segregation and the exponent does not depend on the parameters of the Cahn-Hilliard model. However, the intermittent decay of the segregation indices, before the asymptotic regime is reached, displays a slower algebraic scaling, with exponents down to 1/6 [28], and can even exhibit logarithmic decay, see Fig 2. This intermittent regime can last for several orders of magnitude in time, and an uneven cell type ratio can increase the duration of this regime [28]. Furthermore, while the mobility constant *D* primarily rescales the physical time in the Cahn-Hilliard model, we observe that it can also alter the duration of the intermittent decay. For instance, the simulation displayed in Fig E in S1 Text, which is based on parameters comparable to the ones used for Fig 1 except that the mobility constant *D* is several orders of magnitude bigger, already exhibits an algebraic scaling with the exponent of 1/3 at segregation indices ≤ 1/2.

The determined mobility constants of *D* = 36*μ*m^2^/min and 18*μ*m^2^/min for PFK with EPC and HaCaT with EPC, respectively, are consistent with the range of the experimentally measured mobility constants for each cell type, i.e. PFK (132*μ*m^2^/min), EPC (1.29*μ*m^2^/min) and, HaCaT (1.61*μ*m^2^/min) [20]. In particular, the fitted mobility constant for PFK with EPC is greater than that of HaCaT with EPC, as expected from the individual mobility constants of each cell type.

### Exemplary morphological analysis of both models

**Fig 3:**
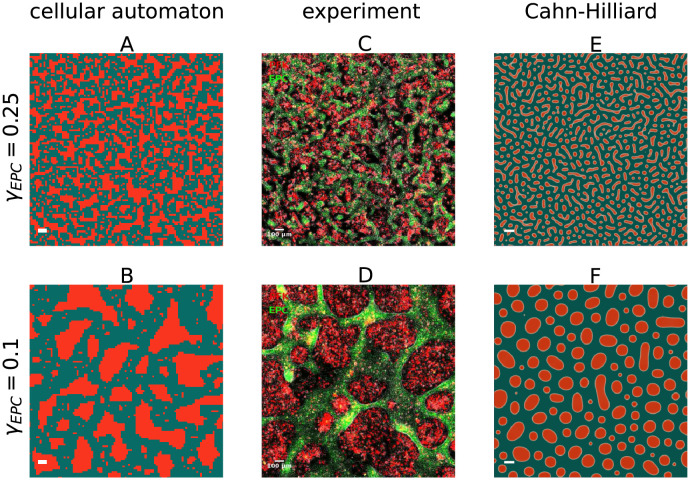
The cellular automaton reproduces morphology and size distribution of the cell clusters in the experiments of Méhes et al. [20] of EPC (green) with PFK (red) closer than the Cahn-Hilliard model. The snapshots of the cell mixtures A, C, E of the first row are taken at a segregation index of EPC *γ*_EPC_ = 0.25, at the start of the experimental recording, while the snapshots B, D, F in the second row are at a segregation index of EPC *γ*_*EPC*_ = 0.1, at the end of the recording. A and B show the cellular automaton, C and D show the experiments and are taken from video S5 in Méhes et al. [20], and E and F show the Cahn-Hilliard model. The snapshots A, B, E and F show a detail from the simulations, such that approximately 100^2^ cells are visible, to match the spatial scale of the snapshots C and D of the experiments. The time points corresponding to the images are marked by black crosses in Figs 1 and 2.

In conclusion, we observe that two fundamentally different models both match the experimental segregation indices on the limited time span, see Figs 1 and 2. Since the segregation indices are not sufficient to distinguish between both models with respect to the experimental observations, we additionally compare the distribution of cluster sizes *ρ*, the morphology of the clusters, and the average cluster diameter at two different levels of segregation qualitatively, see Fig 3: In all three cases, the CA, the CH model and the experiment, the cell type that is less abundant, here PFK shown in red, forms clusters surrounded by a single contiguous domain of the more abundant cell type, here EPC shown in green. The Cahn-Hilliard model results in a rather narrow distribution of cluster sizes while clusters form circular shapes or slightly elongated bulges, see Fig 3E and 3F. In contrast, the cells in the experiment of Méhes et al. [20] display a wider distribution of cluster sizes with different shapes of clusters, see Fig 3C and 3D. Interestingly, the configurations of the cellular automaton exhibit features very similar to the experiment, see Fig 3A and 3B.

**Fig 4:**
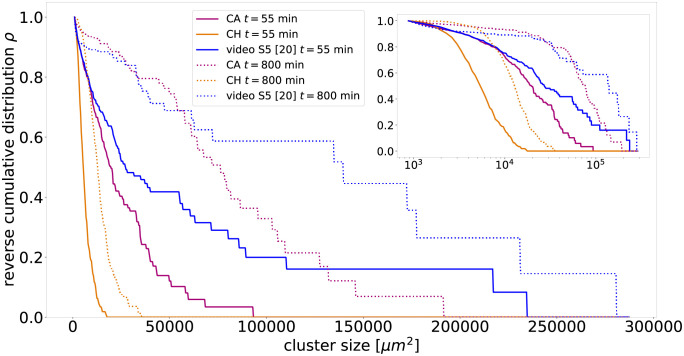
The cellular automaton reproduces the cluster size distribution *ρ* of the experiments of Méhes et al. [20] for EPC with PFK closer than the Cahn-Hilliard model. Shown is the reverse cumulative probability that a randomly drawn cell belongs to a cluster of respective size. For both models and the video S5 from Ref. [20], two separate cluster size distributions are shown, one at an early stage (*t* ≈ 55min) and one at a later stage (*t* ≈ 800min). The cluster size distributions represent exclusively PFK clusters, since EPC as the more abundant cell type forms one large connected sea, which we ignore in the distributions. Note that clusters below 2 cells are neglected as they can not be resolved in the video, see S1 Text.

The results of the qualitative comparison of the cell mixtures of Fig 3 are confirmed by a quanitative analysis of the reverse cumulative distribution of cluster sizes *ρ*, displayed in Fig 4. These distributions are similar between the cellular automaton and the experiment for small cluster sizes. Note, that the cellular automaton exhibits an exponential decay at early times and an algebraic decay with an exponent ≈1 at later times, see also Fig B in S1 Text. In contrast, for the Cahn-Hilliard model, this distribution declines already steeper at roughly an order of magnitude smaller cell sizes than for the cellular automaton model and the experiment. Note that the distribution only represents the PFK clusters, since EPC cells form a single connected cluster. The analysis of the experimental data and the computation of the cluster sizes is detailed in the S1 Text.

**Fig 5:**
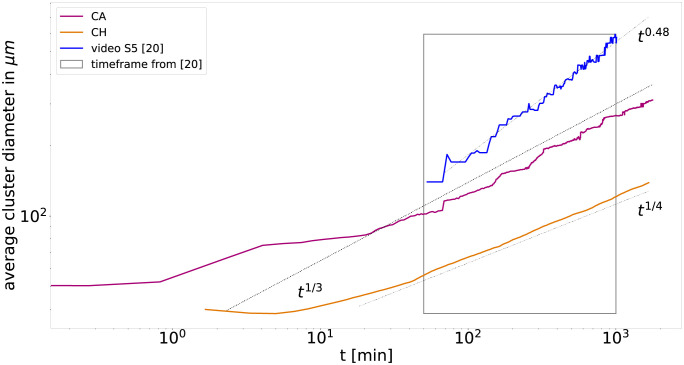
Exemplary comparison of the average cluster diameter in the segregation of PFK and EPC for the cellular automaton model (red line), the Cahn-Hilliard model (orange line) and the experimental data (blue line, based analysis of video S5 of Méhes et al. [20]) computed with two-point correlation method, see Materials and methods. Note that average cluster diameter in both models, cellular automaton and Cahn-Hilliard, are inverse proportional to their segregation indices. In contrast, the average cluster diameter obtained for the experimental data displays a steeper power law than expected from the corresponding segregation indices.

We further use the two point correlation method to obtain the average cluster diameter. Since Méhes et al. [20] report the average cluster diameter of each cell type separately, we reanalyse the experimentally obtained videos to compute the more prevalent cluster diameter of both cell types combined. The comparison of the diameters observed in the models and experiment are displayed in Fig 5. For the models, we obtain an average cluster diameter inverse proportional to the segregation indices with algebraic exponent 1/3 for the cellular automaton and 1/4 for Cahn-Hilliard. In contrast, the experiment shows an even steeper scaling with an algebraic exponent of 0.48.

The differences in the length scale of the average cluster diameter are consistent with the phase images of both models and the experiment, see Fig 3. The Cahn-Hilliard model displays a very narrow cluster size distribution with more smaller clusters in comparison to the cellular automaton and the experiment, which display a much wider distribution with much larger clusters. This results in a shorter characteristic length scale for the Cahn-Hilliard model. Even though the cluster size distributions and the cell segregation indices of the cellular automaton and the experiment are very similar, there are yet significant differences in the length scale and for the scaling over time of the average cluster diameter. We attribute this to the differences in cluster shapes. While clusters appear rounded in the experiment, the clusters in the cellular automaton are still not rounded. This relates to two competing effects in cluster formation, growth of the cluster versus rounding of their interface, and we expect the cluster in the cellular automaton to become rounder on even longer time scales.

The inverse relation between segregation indices and average cluster diameter is consistent with previous CPM models [17, 32]. In contrast, the steeper increase of the experimentally observed cluster diameters with exponent 0.48 > 1/3 means that the average cluster diameter is not inverse-proportional to the segregation indices in this case. Méhes et al. [20] suspected that this is a consequence of collective motion, implying that collective motion contributes to a wider distribution of cluster sizes. Note that, Beatrici et al. [34] studied the effect of collective motion in a segregation model and measured that the algebraic exponent describing the average cluster size increases with introduction of collective motion from 1/2 to 1 (roughly corresponding to exponents 1/4 and 1/2 for the average cluster diameter). In contrast, the average cluster size reported by Krieg et al [8] for the segregation of gastrulating zebrafish embryos cells display a flatter power law with exponent of ≈ 1/5 (roughly corresponding to exponent 1/10 for the average cluster diameter).

### Exemplary fit optimization for two metrics

**Fig 6:**
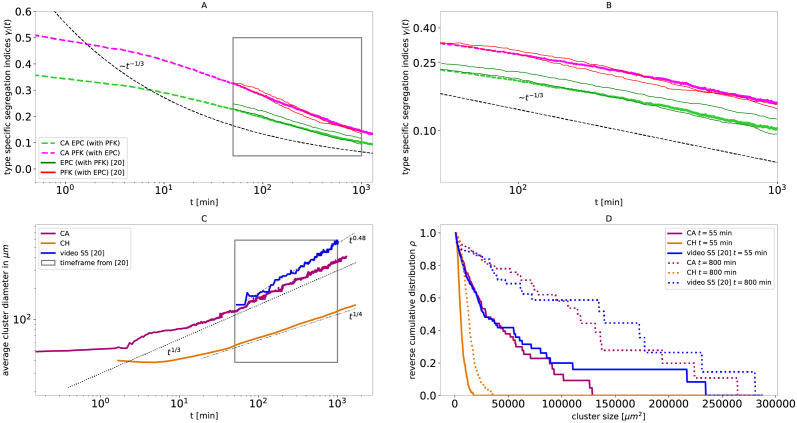
Example representation of the metrics segregation indices *γ*_*i*_, average cluster diameter and cluster size distribution *ρ* with *γ*-*ρ*-fitted parameters for the cellular automaton for the PFK and EPC experiment of Méhes et al. [20]. Panels A and B are analogous to Fig 1, Panel C is analogous to Fig 5 and Panel D is analogous to Fig 4. The simulation used 140^2^ cells with a cell type ratio of *N*_PFK_/*N*_EPC_ = 41.2/58.8, the adhesion parameter (*β*_PFK-PFK_, *β*_EPC-PFK_, *β*_EPC-EPC_) = (−8.0, −5.5, 0.0) and a time scale of migration *τ*_PFK-EPC_ ≈ 20.0 min.

The previously presented metrics, average cluster diameter and cluster size distribution *ρ*, can also be used in the future to improve the fit results of the model. We have done this exemplary for the cellular automaton and the experiment PFK and EPC. As indicated before, several parameters can reproduce the segregation indices similarly well. Thus, the parameter can be further optimized to fit additional metrics, as demonstrated by an exemplary fit of both segregation indices *γ*_*i*_ and cluster size distribution *ρ* in Fig 6. As measures for the goodness-of-fit for the *γ*-*ρ*-fitted parameters, we summarize the averaged mean square deviation Δ*γ* for the segregation indices, see Materials and methods, and the Kolmogorow-Smirnow-distance (KSD) of the cluster size distributions, in Table 1.

**Table 1:** Summary of the averaged mean squared deviation Δ*γ* and the Kolmogorow-Smirnow-Distance (KSD) between each model and the corresponding experiment, see Materials and methods for details.

experiment	model	Δ*γ*[10^−4^]	KSD (*t* = 55min)	KSD (*t* = 800min)
PFK and EPC	CA (*γ*-fitted)	0.642	0.3157	0.4660
PFK and EPC	CA (*γ*-*ρ*-fitted)	0.725	0.1137	0.2393
PFK and EPC	CH	0.939	0.6650	0.8030
HaCaT and EPC	CA (*γ*-fitted)	1.104		
HaCaT and EPC	CH	2.806		

**Fig 7:**
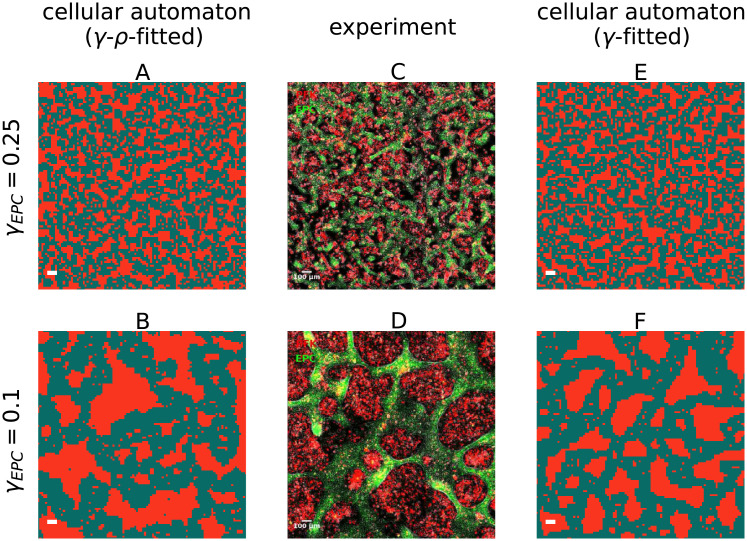
The cellular automaton with *γ*-*ρ*-fitted parameters reproduces the morphology and size distribution of the cell clusters *ρ* in the experiments of Méhes et al. [20] of EPC (green) with PFK (red) closer than the cellular automaton with the *γ*-fitted parameters. The snapshots of the cell mixtures A, C, E of the first row are taken at a segregation index of EPC *γ*_EPC_ = 0.25, at the start of the experimental recording, while the snapshots B, D, F in the second row are at a segregation index of EPC *γ*_*EPC*_ = 0.1, at the end of the recording. A and B show the cellular automaton with optimised parameters, C and D show the experiments and are taken from video S5 in Méhes et al. [20], and E and F show the cellular automaton with the Δ*γ* fitted parameters. The snapshots A, B, E and F show a detail from the simulations, such that approximately 100^2^ cells are visible, to match the spatial scale of the snapshots C and D of the experiments.

The averaged mean square deviation shows, that the cellular automaton reproduces in any case the experimental segregation indices better than the Cahn-Hilliard model. Both parameter fits for the CA reproduce the segregation indices of the experiment well. Further, the calculated KSD shows that the *γ*-*ρ*-fitted parameters of the cellular automaton reproduce the cluster sizes of the experiment much better. Exemplary configurations for the two parameter fits are compared to the experimental observations in Fig 7.

### Parameter influence of the cellular automaton on the segregation

We have already shown that in the segregating experiments the pseudo-algebraic scaling can be explained both by the transitory logarithmic scaling from the cellular automaton and by the transitory algebraic scaling with exponent of 1/4 from the Cahn-Hilliard model. Yet, despite the fact that both models only incorporate adhesion forces, as proposed by Steinberg, the resulting segregation differs fundamentally between both models. In addition, in the time frame of the experiment neither model generates an algebraic scaling with an exponent 1/3, which is usually associated with fluid-like segregation. Firstly this highlights, that not only an algebraic exponent of 1/3 corresponds to fluid-like segregation, but exponents between 1/6 and 1/3 may indicate it as well. Secondly, this implies that in contrast to implicit suggestions of previous works, an exponent differing from 1/3 does not necessitate other intercellular interactions or mechanical forces besides adhesion. In particular, the scaling law with exponent of 1/3 only applies to the asymptotic regime of the models. In contrast, both the Cahn-Hilliard model and the cellular automaton reproduce the experimental data not in the asymptotic but in their respective transitory regime, during which the scaling behavior is more complex and versatile. Additionally this implies, that the transitory regime of the models has a greater relevance for biological cell segregation processes than the asymptotic one.

**Fig 8:**
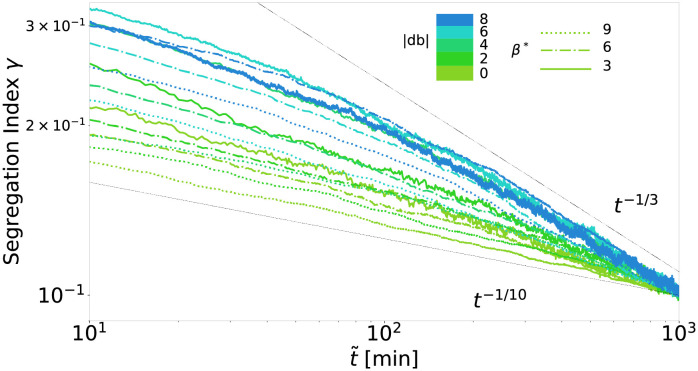
A 1/3 exponent forms an upper bound for the pseudo-algebraic scaling in cellular automaton. Segregation indices obtained from the simulation are shown for a range of the effective parameters *db* and β*, but only for the last two orders of magnitudes in time $\tilde{t}$ before the segregation index reaches *γ* = 0.1 in each simulation. For comparability, the time scale of each simulation is set such that all simulations reach *γ* = 0.1 at $\tilde{t}=1$. For each simulation we use 100^2^ cells, a cell type ratio of 50/50, periodic boundary conditions, and a random mixture *γ* = 0.5 as initial configuration.

To relate the range of segregation dynamics displayed by the cellular automaton to previous experiments, we study numerically the pseudo-algebraic scaling exponents, which can be generated by the automaton, and how they depend on the adhesion parameters. The effective adhesion parameters *db* and β* determine the kinetics of the segregation results and therefore, by adjusting these parameters, we are able to study the impact of those on the possible exponents. The exact influence of *db* and β* on the scaling behavior is complex [42]. The cellular automaton is capable of producing a wide range of pseudo-algebraic scalings, see Fig 8. The scaling behavior changes within the experimental regime of segregation indices and is thus transitory for all displayed parameter choices. Even the flattest curve close to *t*^−1/10^ clearly shows this behavior on longer time scales, see Fig I in S1 Text. We observe an upper bound for the exponent of the pseudo-algebraic scaling at 1/3, consistent with asymptotic exponents observed in previous particle models [16–18, 31, 33]. Due to the logarithmic decay, the pseudo-algebraic scaling exponent over two orders of magnitudes increased with increasing starting time of the observation window, i.e., it is maximal if the segregation indices at the start of the observation are small.

**Fig 9:**
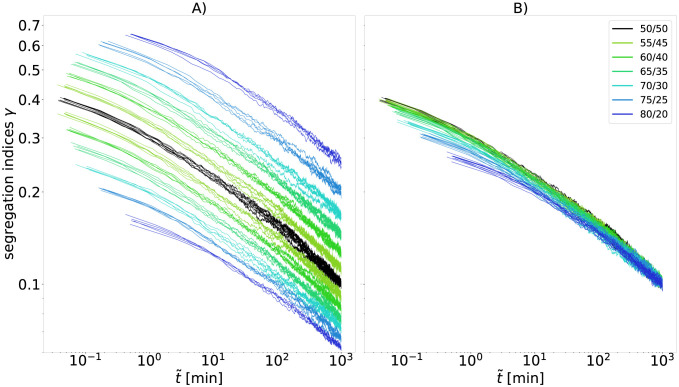
Segregation indices obtained from the simulation shown for a range of cell type ratios. For each simulation we use 100^2^ cells, periodic boundary conditions, *db* = 0, β* = 3, and a random mixture as initial configuration. For comparability, the time scale of migration *τ* of each simulation is set such that all simulations reach segregation indices *γ*_0_ and *γ*_1_ with *γ*_0_*N*_0_ = *γ*_1_*N*_1_ = 500 at dimensionless time $\tilde{t}=1$. Every color represents a specific cell type ratio, while each cell type ratio was simulated five times. Panel A shows the raw data of the simulations. The black lines correspond to an even cell type ratio, for which both segregation indices match, while for uneven ratios the segregation index of the more abundant cell type is below the black line and the other above. Panel B shows the same data where each segregation index *γ*_*i*_ is rescaled to a segregation index ${\tilde{\gamma }}_{i}$ at an even ratio according to ${\tilde{\gamma }}_{0}=2\gamma_{0}N_{0}/\left(N_{1}+N_{0}\right)$.

However, in contrast to the parameters *db* and β*, the cell type ratio does not influence the scaling, thus also not the pseudo-algebraic exponents, which is consistent with recent observations in the CPM model [32]. As shown in Materials and Methods and visualized in Fig 9A, the cell type ratio just increases the distance between *γ*_0_ and *γ*_1_, but never the slope in the last orders of magnitudes in time, Fig 9B.

## Discussion

We reproduce the experimentally observed segregation indices of Méhes et al. [20] by a 2D cellular automaton model which solely incorporates differential adhesion. The parameters of the model are calibrated according to the experimental setups. For the calibration, an efficient algorithm is developed which makes the large number of simulations required for the exploration of the parameter space feasible. While Méhes et al. interpreted the decay of the experimental segregation indices as an algebraic scaling with the exponent of 1/3, the cellular automaton model exhibits a logarithmic decay at the time scale of the experiment, which corresponds to the transitory regime of the model. We attribute this contradiction to the limited time span observable in the experiment, which is insufficient to determine the scaling of the segregation indices. Thus, we refer to the seemingly algebraic decay observed on a limited time span as pseudo-algebraic scaling. The match of the experimental results and the ones from the cellular automaton highlights the possible ambiguity of scalings on short time spans. We quantify the range of exponents possible with the pseudo-algebraic scaling of the cellular automaton model and find the exponent of 1/3 to be an upper bound. Since an algebraic decay of the segregation indices with exponent 1/3 is commonly considered for fluid-like segregation, and Steinberg [14] proposed that cell segregation is similar to that of fluids, we additionally compare the experimental results with fluid segregation expressed by the 2D Cahn-Hilliard model. In order to adjust the spatial scale of the Cahn-Hilliard model to the cell segregation experiment, we developed a mapping between the cellular automaton and the Cahn-Hilliard model. The resulting segregation indices from the Cahn-Hilliard model fit well the experimental ones, although they rather follow an pseudo-algebraic decay with exponent 1/4 than 1/3 on the relevant time interval, which is again hard to distinguish on the short time span of the experimental data.

Note that the Cahn-Hilliard model, which well describes fluid segregation in a diffusive regime, displays an algebraic decay of segregation indices with exponent 1/3 only asymptotically. There is also an intermittent regime, which can last several orders of magnitude in time, during which exponents down to 1/6 are possible [28]. The smaller exponent of 1/4 observed by us means that for the setup of the experiment the corresponding fluid-like segregation dynamics are in the intermittent regime. On the one hand, this highlights that experimentally observed exponents smaller than 1/3 do not necessarily rule out fluid like segregation. On the other hand, this demonstrates the importance of calibrating segregation models to actual experimental data, as only limited time regimes of the model may be experimentally relevant.

In conclusion, the calibration of both models, the cellular automaton and the Cahn-Hilliard model, to the experimental setup reveals that the transitory regime of these models is relevant for the spatio-temporal scales of the experiment rather than the asymptotic regime. This is in contrast to most of the theoretical studies, which usually focus on the asymptotic regime of the cell segregation models and do not calibrate the model parameters to the physical constraints of the experiments. It is reasonable to expect that also for the CPM the experimental data falls in the transitory regime due to analogies between segregation processes in the cellular automaton model and the CPM, especially the analogous structure of the exponent of the cell switch rates in the cellular automaton and the energy functional in the CPM. Only recently, asymptotic cell segregation in the CPM has been explained by directly applying effective adhesion parameters, a concept previously studied in cellular automata [5, 42]. Our findings suggest that future studies on theoretical models and corresponding numerical simulations of cell segregation should examine not only the asymptotic regime, but also the complex and less understood kinetics of the transitory regime.

We present a way to fit both models to experimental data, which can be applied to future experiments. Since the cell type ratio can directly obtained from the segregation indices ratio and the time scale of migration rescales the time scale by a factor, only two parameters remain to be fitted for the cellular automaton. Note that our calibration approach should be applicable to other cell-based models, including the CPM. With respect to the mapping we developed between the Cahn-Hilliard model and the cellular automaton model, only the mobility constant *D* as a single parameter has to be fitted for the Cahn-Hilliard model.

### Issues with scaling analysis

We point out that in experiments only two or less orders of magnitudes in time are available to determine the scaling behavior [8, 20, 38]. Our results suggest, that scaling behavior of segregation indices on such short time spans is ambiguous, and algebraic scaling on these time spans should be rather called pseudo-algebraic scaling, since it may also be a misinterpreted logarithmic decay. This possible ambiguity has already been hinted at before: Nakajima and Ishihara mentioned, that their segregation can also be interpreted as a logarithmic scaling since the algebraic decay was measured only in the last orders of magnitude [17]. Belmonte et al. indicated that a logarithmic decay might be possible, if no coordinated motion of neighbor cells is present [16]. We show that the cellular automaton, which is solely based on differential adhesion, can generate pseudo-algebraic decays which cover the same range of exponents ≤ 1/3 as models which additionally incorporate other mechanisms like collective motion or differential velocities [16–18, 31]. This wide range of possible segregation behavior is a feature of the transitory regime while we observe no steeper scaling than *t*^1/3^. This puts a new perspective on conclusions of previous studies, which focused mainly on the asymptotic behavior of segregation models. In particular, this implies that deviations of biological segregation processes from the algebraic scaling with exponent 1/3 do not rule out that the segregation is solely based on the minimization of the total surface energy. In conclusion, due to the ambiguity in the transitory regime and for short observation spans, it is not possbile to distinguish between specific models and thus to determine which mechanisms govern the segregation solely based on the scaling behavior.

Many studies infer from the scaling behavior of the segregation indices the impact of certain cell mechanisms, like collective motion on, cell segregation. In contrast, our results strongly suggest utilizing additional metrics of segregation when comparing between simulations and experiments to overcome the ambiguous interpretations of the segregation indices of experimental data on limited time spans. Such segregation metrics could be the cluster size distribution *ρ*, the morphology of the clusters, and the average cluster diameter. As an example of such an analysis, we compute the cluster size distribution and the average cluster diameters for PFK with EPC and compare them between models and experiment. We find that the cellular automaton does not only reproduce the segregation indices, but also has a more similar cluster size distribution compared to the experiment, in contrast to the Cahn-Hilliard model, which misses the large clusters that are present in the cellular automaton and the experiment. On the other hand, the average cluster diameter differs between the models and the experiment. For the models, we obtain, as expected, an average cluster diameter inverse proportional to the segregation indices with algebraic exponent 1/3 for the cellular automaton and 1/4 for Cahn-Hilliard. In contrast, the experiments display a steeper algebraic scaling with exponent 0.48, meaning that the average cluster diameter is not inverse proportional to the segregation indices, which has been attributed to collective motion [20]. In conclusion, the cellular automaton reproduces the experimental cell segregation better than the Cahn-Hilliard model, but still misses features which may be related to collective motion, but are not incorporated in the model. In fact, the similarities between experiment and cellular automaton in the cluster size distribution *ρ* and the segregation indices *γ*_*i*_ together with the differences in the average cluster diameter point towards differences in cluster shapes between model and experiment. Note that all models display scaling behavior consistent with the experimental one. Only by considering additional metrics, in our case the cluster size distribution, and directly comparing the corresponding time series between experiment and the calibrated models, a distinction between the models becomes possible.

Note, that the segregation in the cellular automaton follows the diffusion-and-coalescence mechanism [41]. In particular, the diffusion of clusters is driven by fluctuations of cells at the clusters’ boundaries, see also the exemplary video S1 Movie in S1 Text. The diffusion-and-coalescence mechanism is usually associated asymptotically with an algebraic scaling with exponent 1/4 [17, 32, 34, 35, 41], reflecting the competition between the two effects driving segregation: the growth of clusters versus the rounding of their interfaces. However, at the intermediate time scales considered here the clusters in the cellular automaton are not sufficiently rounded yet, which most likely causes a steeper scaling with exponent 1/3.

### Additional observations

Note that the inverse relation between segregation indices and average cluster diameter is consistent with observations in previous CPM models [17, 32]. In addition the range of exponents observed in our cellular automaton model is consistent with previous models of 2D cell segregation without collective motion [17, 18, 32, 34, 35], while the addition of collective motion accelerates segregation leading to larger exponents [16, 34]. The biggest difference between the cellular automaton and the experiment is the steeper increase of the cluster diameter in the experiment with exponent 0.48. Note, however, that cluster sizes reported by Krieg et al [8] for the segregation of gastrulating zebrafish embryos cells display a much flatter power law with exponent of ≈ 1/5 (roughly corresponding to exponent 1/10 for the average cluster diameter).

Another interesting feature of cell segregation is which cell type encloses the other. While it seems reasonable that the more abundant cell type should enclose the other, Beatrici and Brunnet [18] have found different behavior depending on the cell type ratios and the cells’ velocities. In addition, to resolve the contradicting logarithmic decay found by Glazier and Graner [13] in the CPM and the algebraic scalings found in successive studies with CPM [17] and particle models [16, 18], Nakajima and Ishihara [17] proposed that the number of cells considered in a simulation affects the scaling behavior. Our results suggest that rather the time regime determines the scaling exponents observed over one or two orders of magnitudes. This is consistent with the fact that many simulations display a logarithmic decay initially, independently of the number of cells [16–18, 38]. This is further supported by Beatrici and Brunnet [18], which found no difference in the scaling behavior for a wide range of cell numbers (500 to 8000) in their simulations. Recently Durand [32] also questioned the effect of the numbers of cells on the scaling behavior. Likewise, we observe the same logarithmic decay for a range of 25^2^ to 140^2^ cells per simulation, while only the fluctuations of the segregation indices are diminished by using more cells.

## Materials and methods

### Cellular automaton: Model and calibration

For simulating cell segregation, we use a cellular automaton based on Voss-Böhme et al. [19]. We use a 2D-quadratic lattice *S* with N∈N nodes for each dimension, *S* = {1, …, *N*} × {1, …, *N*}. We assign exactly one cell to each node. Each cell has the area of (Δ*x*)^2^, which leads to lattice lengths *N*Δ*x* for each side. Every cell is mapped to a specific cell type *W* = {0, 1} with *ξ* : *S* → *W* defining a specific configuration of cells on the lattice. Based on two possible cell types, we define three adhesion parameters ***β*** = (*β*_11_, *β*_10_, *β*_00_)^*T*^ which set the stickiness of two directly neighboring cells depending of their type. The more two neighboring cells stick to each other, the larger the associated *β*_*ij*_ parameter. *τ* denotes a parameter to adjust the time scale of migration in the simulation. Further, based on these parameters, the rate *r*(**x**, **y**) of two cells at neighboring positions **x**, **y** ∈ *S*, |**x** − **y**| = Δ*x* swapping their locations is given by:

$$\begin{array}{c} r\left(x,y,\xi \right)=\{\begin{array}{cc} \tau^{-1}\text{exp}\;\left\{\beta_{\text{sum}}\left(x,y,\xi \right)\right\} & \text{,}\;\text{if}\;\xi (y)\neq \xi (x)\;\text{and}\;|x-y|=\Delta x\\ 0 & \text{,}\;\text{otherwise} \end{array} \end{array}$$ (1)

where

$$\begin{array}{c} \beta_{\text{sum}}\left(x,y,\xi \right)=-\sum_{z:|z-x|=\Delta x}^{}\beta_{\xi (x)\xi (z)}-\sum_{z:|z-y|=\Delta x}^{}\beta_{\xi (y)\xi (z)}\text{.} \end{array}$$ (2)

Notice that the definition of the homotypic adhesion parameters in Eqs (1) and (2) is such that smaller (or more negative) parameters lead to higher migration rates and therefore represent lower adhesion forces. Instead of using the usual Metropolis algorithm and Monte-Carlo steps, this model is implemented in continuous time by applying the idea of the Gillespie algorithm to the cellular automaton, see S1 Text for details, which results in a speed-up of the simulations by several orders of magnitude.

Further, the cellular automaton simulates segregation and thus the segregation indices $\gamma (\tilde{t})$ in a dimensionless time $\tilde{t}$. The time scale of migration *τ* which transforms this dimensionless time $\tilde{t}$ into physical time *t*, as $t=\tau \tilde{t}$, is calibrated based on the experimental data. By matching the physical time $t_{\gamma =\gamma_{\text{match}}}$ at which the experimental segregation indices first reaches a particular value *γ*_match_, such that $\gamma_{\text{exp}}\left(t_{\gamma =\gamma_{\text{match}}}\right)=\gamma_{\text{match}}$ and the dimensionless time ${\tilde{t}}_{\gamma =\gamma_{\text{match}}}$ at which the simulated segregation indices first reaches this value $\gamma_{\text{sim}}\left({\tilde{t}}_{\gamma =\gamma_{\text{match}}}\right)=\gamma_{\text{match}}$, and estimate $\tau =t_{\gamma =\gamma_{\text{match}}}/{\tilde{t}}_{\gamma =\gamma_{\text{match}}}$. If no value for *τ* is provided, it is set to 1 dimensionless and therefore neglected.

Voss-Böhme et al. [19] proposed an effective parameter β* for two cell types, which determines the asymptotic sorting behavior, where

$$\begin{array}{c} \beta^{*}=\beta_{00}+\beta_{11}-2\beta_{10}\text{.} \end{array}$$ (3)

The impact of this parameter has been numerically confirmed and generalized to an arbitrary number of cell types by Rossbach et al. [42]. We reparametrize the adhesion parameters ***β*** based on the effective parameter β* to better describe the impact of the parameters on the segregation behavior:

$$\begin{array}{c} db=\beta_{11}-\beta_{00}\text{,} \end{array}$$ (4)

$$\begin{array}{c} d=\beta_{00}+\beta_{10}+\beta_{11}\text{.} \end{array}$$ (5)

This leads to the following invertible transformation equation:

$$\begin{array}{c} \beta =(\begin{array}{c} \beta_{00}\\ \beta_{10}\\ \beta_{11} \end{array})=\frac{\beta^{*}}{3}(\begin{array}{c} \frac{1}{2}\\ -1\\ \frac{1}{2} \end{array})+\frac{d}{3}(\begin{array}{c} 1\\ 1\\ 1 \end{array})+\frac{db}{2}(\begin{array}{c} -1\\ 0\\ 1 \end{array})\text{.} \end{array}$$ (6)

The parameter *d* rescales the rates in a trivial way, since an increase of *d* by Δ*d* will increase all *β*_*ij*_ by the same amount 1/3Δ*d* and therefore decrease all rates by a factor exp{−8/3Δ*d*}, independently of *ξ*, *x*, *y*:

$$\begin{array}{c} \begin{array}{cc} r(x,y) & =\tau^{-1}\text{exp}\;\{-\sum_{z:|z-x|=\Delta x}(\beta_{\xi (x)\xi (z)}+\frac{1}{3}\Delta d)-\sum_{z:|z-y|=\Delta x}(\beta_{\xi (y)\xi (z)}+\frac{1}{3}\Delta d)\}\\ & =\tau^{-1}\text{exp}\;\{-\frac{8}{3}\Delta d\}\;\text{exp}\;\{-\sum_{z:|z-x|=\Delta x}\beta_{\xi (x)\xi (z)}-\sum_{z:|z-y|=\Delta x}\beta_{\xi (y)\xi (z)}\} \end{array}\text{.} \end{array}$$ (7)

The factor exp{−8/3Δ*d*} just rescales the time scale of migration *τ*.

**Fig 10:**
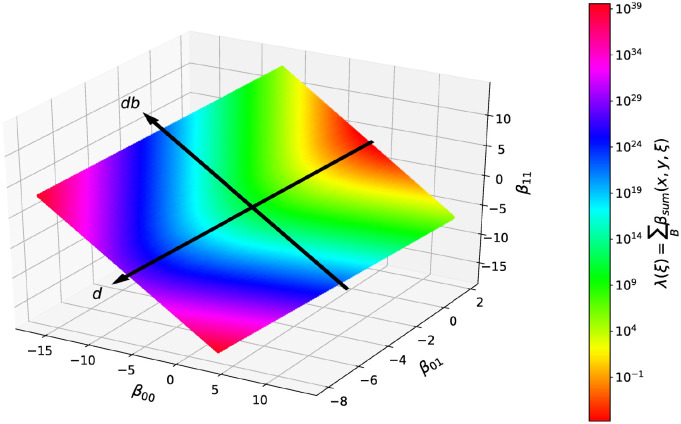
The influence on the time scale is trival for the parameter *d*, and non trival for the parameter *db*. Shown is, for a constant β* = 3 and random initial conditions *ξ* with a 50/50 cell type ratio, the color coded sum λ_0_ of all heterotypic transitions rates. In direction of (1, 1, 1)^*T*^, the value of λ decreases and therefore the simulation time Δ*t*_swap_ for two neighboring heterotypic cells to change positions increases. In direction of (−1, 0, 1)^*T*^, the time dependency is nontrivial, but symmetric to *db* = 0.

The effects of the parameter *db* on the model system are more complex and have been examined numerically. We initialize with a random configuration *ξ* and measure successively, for each subsequent configuration *ξ*_*t*_ the sum λ_*t*_ of all heterotypic transition rates in the whole system at this time. The value λ_*t*_ sets the current average waiting time Δ*t*_swap_ = 1/λ_*t*_ between two cell switches, see implementation of the cellular automaton in S1 Text. We find that on average an increase of the parameter *db* will increase λ_*t*_ and therefore decrease the average waiting time Δ*t*_swap_. As illustration we show the dependency of λ_0_ on the parameters for a random configuration *ξ* in Fig 10. Further, for a fixed parameter β*, an increase of *db* will also increase the computing time, i.e., the number of cell switches required to reach the same level of segregation [42].

To further assess the influence of the initial configurations *ξ*, we generated configuration with segregation indices 0.25, as observed initially in the experiments, by evolving a randomly mixed configuration with different adhesion parameter sets up to this point and then changed the adhesion parameters for further time. The comparison between the segregation processes for the same adhesion parameters but the different initial conditions, displayed in in Fig K in S1 Text, reveals a small influence of the initial condition, but the scaling of the segregation indices is not affected.

### Segregation index

As in the experiment, we use type specific segregation indices *γ*_*i*_ to determine the degree of segregation over time in the cell-based model. For type *i* ∈ *W*, the index *γ*_*i*_ is the average of the amount *n*_≠_(*k*) of heterotypic neighbors, where the average is taken over all positions *k* carrying cells of type *i*, in relation to the maximum possible numbers of neighbors, which is 4 for a von-Neumann neighborhood,

$$\begin{array}{c} \gamma_{i}=\frac{1}{4}{\left\langle n_{\neq }\left(k\right)\right\rangle }_{k\in S\eta (k)=i}=\frac{1}{4N_{i}}\sum_{k\in S\;\eta (k)=i}n_{\neq }\left(k\right)=\frac{1}{4N_{i}}I\text{,} \end{array}$$ (8)

where *N*_*i*_ denotes the total number of cells of type *i* and *I* denotes the interface length,

$$\begin{array}{c} I=\sum_{k\in S\;\eta (k)=i}n_{\neq }\left(k\right)\text{,} \end{array}$$ (9)

which is another commonly used measure of segregation. Further, if an even cell type ratio is given (50/50), it applies *N*_*i*_ = *N*^2^/2, where *N*^2^ = |*S*|. The resulting prefactor 2*N*^2^ is equal to the maximum achievable interface length in the cellular automaton, which corresponds to a checkerboard configuration where each cell has four heterotypic neighbors, *I*_max_ = 2*N*^2^. Based on this, the relative interface length *I*_*r*_ can be defined as the interface length *I* normalized by the maximal interface length *I*_max_,

$$\begin{array}{c} I_{r}=\frac{I}{I_{\text{max}}}=\frac{1}{2N^{2}}\sum_{k\in S\;\eta (k)=i}n_{\neq }\left(k\right)\text{.} \end{array}$$ (10)

Thus, for an even cell type ratio *N*_0_ = *N*_1_ the relative interface length *I*_*r*_ is equal to the segregation indices *γ*_0_ = *γ*_1_ = *I*_*r*_. If the numbers of cells of each type *N*_*i*_ are not equal, it follows from Eq (8) that the segregation indices *γ*_*i*_ are inverse-proportional to the cell type ratio

$$\begin{array}{c} \frac{\gamma_{0}\left(t\right)}{\gamma_{1}\left(t\right)}=\frac{N_{1}}{N_{0}}\text{.} \end{array}$$ (11)

Therefore, the scaling exponents of *γ*_*i*_ and *I*_*r*_ are always identical.

Based on Eq (8) it is possible to calculate the minimal segregation indices for a given field *N*^2^. Since we only use a quadratic field with periodic boundary condition for our simulations, the minimal interface length can be assumed to be *I*_min_ ≈ 2*N*. For the corresponding segregation indices it follows:

$$\begin{array}{c} \gamma_{\text{i,min}}=\frac{1}{4N_{i}}I_{\text{min}}\approx \frac{N}{2N_{i}}\text{.} \end{array}$$ (12)

Note, that for equal cell ratio $N_{i}=\frac{N^{2}}{2}$ this lower boundary scales inversely with the system size *γ*_i,min_ ≈ *N*^−1^.

To determine the goodness-of-fit for the segregation indices, we calculate the averaged mean squared deviation with the following algorithm:

1. Choose 50 time points evenly on a logarithmic scale within the relevant time interval.
2. Determine the corresponding values of the experimental segregation indices by piecewise linear interpolation between the discrete observation points of the experiment.
3. For each cell type, HaCaT, PFK or EPC, average the two experimental time series evaluated at the above chosen 50 time points.
4. Determine the squared deviation per point of the averaged experimental data and the corresponding value of the simulation.
5. Average the squared deviation over both cell types for the each experiment.

The previous equations Eqs (8) and (11) apply exactly for periodic boundary conditions. For other boundary conditions, the cell type ratio still approximates the type specific segregation indices ratio *γ*_0_(*t*)/*γ*_1_(*t*) ∼ *N*_1_/*N*_0_, and the segregation indices approximate the relative interface length *I*_*r*_ ∼ *γ*_*i*_. This is due to the fact that boundary cells at the edge and in the corners have less than 4 neighbors, but their contribution gets less with rising lattice size *N*, since the boundary size scales with *O*(*N*) and the lattice size scales with *O*(*N*^2^).

**Fig 11:**
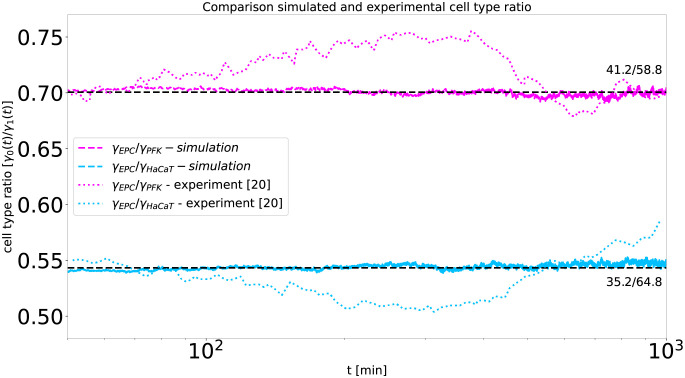
The cell type ratio for the simulation can be obtained from the experiments of Méhes et al. [20]. Shown is a comparison of the cell type ratio *r* = *γ*_0_(*t*)/*γ*_1_(*t*) from the experiments of Méhes et al. [20] (dotted in color) and from the cellular automaton (dashed in corresponding color). The cell type ratio in the cellular automaton is set to the approximate mean of the ratios observed in the experiment (dashed black lines). The cell type ratio was calculated by the ratio of the type specific segregation indices for each time *t* per experiment EPC with PFK (red) and HaCaT with EPC (blue).

Based on Eq (11) it is possible to calculate for every pair of type specific segregation indices *γ*_0_(*t*) and *γ*_1_(*t*) the corresponding cell type ratio and vice versa. We define the cell type ratio as

$$\begin{array}{c} r≔\frac{\gamma_{0}}{\gamma_{1}}\;\text{with}\;N_{0}\geq N_{1}\text{,} \end{array}$$ (13)

where we assume without loss of generality that *r* ≤ 1.

In this sense, the cell type ratio in the experiment can be obtained from the ratio of the corresponding segregation indices, see Fig 11. Indeed, the ratio of segregation indices is relatively constant over the time span of the experiment, and we set the cell type ratio of the cellular automaton accordingly, as indicated in Fig 11. In their experiments, Méhes et al. [20] choose the cell type ratio such that initially the same amount of area is covered by each type. Since the cells of each type are similar in size, as EPC is 300*μm*^2^ [43], PFK is 400*μm*^2^ [15] and HaCaT is 80–400*μm*^2^ [44], it is reasonable to simulate the segregation with the cellular automaton, where every cell has the same space of the grid. However, the small differences in size imply that the number of cells of each type in the experiment is not equal. Instead, one estimates from the cell size ratios 0.75 = *A*_EPC_/*A*_PFK_ = *A*_EPC_/*A*_HaCaT_, cell type ratios which are consistent with the ones obtained from the ratio of segregation indices 0.70 = *N*_PFK_/*N*_EPC_ and 0.54 = *N*_HaCaT_/*N*_EPC_ for EPC with PFK and HaCaT with EPC, respectively, see Fig 11. Note that the specific cell type sizes where not reported by Méhes et al. [20] and cells can vary in size during an experiment as well as depending on the experimental setup.

Additionally to the segregation indices and the interface length, the average cluster diameter is a third commonly used measure to determine order in segregation processes. For the cellular automaton it can be shown that the average cluster diameter *d* is inverse-proportional to the interface length *I*, assuming the cell type ratio equals 50/50, the cluster size distribution is narrow, i.e. ${\langle d_{l}\rangle }^{2}\approx \langle d_{l}^{2}\rangle$, the total area *A*_sum_ of all clusters is constant, and the clusters are approximately circular. In the following *n*_*c*_ denotes the number of clusters, $A_{l},l\in N|1\leq l\leq n_{c}$ the size and *U*_*l*_ the scope of the *l*-th cluster. Approximating the clusters as circles, we have

$$\begin{array}{c} \begin{array}{cc} & \sum_{l}^{n_{c}}A_{l}=A_{\text{sum}}=n_{c}\left\langle A_{l}\right\rangle =n_{c}\frac{4}{\pi }\left\langle d_{l}^{2}\right\rangle \Leftrightarrow n_{c}=\frac{4A_{\text{sum}}}{\pi \langle d_{l}^{2}\rangle }\\ & \sum_{l}^{n_{c}}U_{l}=I=n_{c}\left\langle U_{l}\right\rangle =n_{c}\pi \left\langle d_{l}\right\rangle \Leftrightarrow n_{c}=\frac{I}{\langle d_{l}\rangle \pi }\text{.} \end{array} \end{array}$$ (14)

By combining the two expressions for *n*_*c*_ in Eq (14), we get

$$\begin{array}{c} \left\langle d_{l}\right\rangle =\frac{I\langle d_{l}^{2}\rangle }{4A_{\text{sum}}}\Rightarrow \left\langle d_{l}\right\rangle \underset{{\left\langle d_{l}\right\rangle }^{2}\approx \left\langle d_{l}^{2}\right\rangle }{\approx }\frac{\left\langle d_{l}^{2}\right\rangle }{\left\langle d_{l}\right\rangle }=\frac{4A_{\text{sum}}}{I}\text{,} \end{array}$$ (15)

where the last approximation is only valid for a narrow distribution of cluster sizes. Since 4*A*_sum_ is constant, it results that the average cluster diameter is inverse-proportional to the interface length 〈*d*_*l*_〉 ∼ 1/*I*. We infer from the fact that the average cluster diameter in both, the cellular automaton and the Cahn-Hilliard model, is inverse-proportional to the interface length, that their distribution of cluster sizes is sufficiently narrow. This is consistent with recent observations for the CPM [32], where the same inverse-proportional behavior is observed asymptotically when the formed clusters are approximately circular.

## Conclusion

By calibrating a 2D cellular automaton model which solely incorporates differential adhesion to the experimental setup of Méhes et al. [20], we reproduce experimentally observed segregation indices. While Méhes et al. interpreted the decay of the experimental segregation indices as an algebraic scaling with the exponent of 1/3, the cellular automaton model exhibits a logarithmic decay at the time scale of the experiment, as it belongs to the transitory regime of the model. Since Steinberg [14] also proposed that cell segregation is similar to that of fluids, we additionally compare the experimental results with fluid segregation expressed by the 2D Cahn-Hilliard model. By developing a mapping between the cellular automaton model and the Cahn-Hilliard model, only one parameter remains to be fitted. The resulting segregation indices from the Cahn-Hilliard model fit the experimental ones well, although they rather follow an pseudo-algebraic decay with exponent 1/4 than 1/3 on the relevant time interval. The match of the experimental results with both models highlights the possible ambiguity of scalings on the short time spans of the experimental data. Our results also emphasize that the transitory regime of these models is relevant for the spatio-temporal scales of the experiment rather than the asymptotic regime. This is in contrast to most of the theoretical studies, which usually focus on the asymptotic regime of the cell segregation models and do not calibrate the model parameters to the physical constraints of the experiments.

Our results highlight the importance of additional metrics to compare segregation between simulations and experiments, in order to avoid the ambiguity of scaling laws on the limited time spans of the experiments. Thus, future experiments on cell segregation should report their observations in terms of several metrics, like segregation indices, cluster size distribution and average cluster diameter, and provide the raw data to allow further retroactive analysis in comparison with simulations.

While our focus here is segregation in 2D experiments and models, it would be interesting to extent our approach to 3D tissues. Cochet-Escartin et al. [38] studied segregation in 3D tissue over half an order of magnitude of time. They measured an algebraic decay with exponent 0.74 for the segregation indices in the experiment and 1/2 for that in a corresponding CPM model. Note that the measured algebraic decay is only displayed for a quarter order of magnitude in time. However, this is remarkable, since the exponent of the algebraic decay in a 3D space should rather decrease, compared to a 2D space according to the diffusion-coalescence mechanism [34, 45, 46]. This discrepancy suggests that the segregation was observed in the transitory regime, which points to the importance of studying transitory regimes in 3D tissues as well.

## Supporting information

S1 Text Supporting figures and tables. The Supporting Information S1 Text provides details on the cellular automaton implementation, the Cahn-Hilliard model and its mapping to the cellular automaton, as well as the background of the used 2D-image analysis methods. In addition it contains supporting figures to strengthen our findings.
(PDF)

S1 Movie Supporting information S1 Movie provides an exemplary illustration of cellular automaton segregation. (GIF)

## References

[1] Townes PL, Holtfreter J. Directed movements and selective adhesion of embryonic amphibian cells. J Exp Zool. 1955;128(1):53–120. doi: 10.1002/jez.1401280105 15559931

[2] Cerchiari AE, Garbe JC, Jee NY, Todhunter ME, Broaders KE, Peehl DM, et al. A strategy for tissue self-organization that is robust to cellular heterogeneity and plasticity. Proc Natl Acad Sci USA. 2015;112(7):2287–2292. doi: 10.1073/pnas.1410776112 25633040 PMC4343104

[3] Xiong F, Tentner AR, Huang P, Gelas A, Mosaliganti KR, Souhait L, et al. Specified neural progenitors sort to form sharp domains after noisy Shh signaling. Cell J. 2013;153(3):550–561. doi: 10.1016/j.cell.2013.03.023 23622240 PMC3674856

[4] Kay RR, Thompson CRL. Forming Patterns in Development without Morphogen Gradients: Scattered Differentiation and Sorting Out. Cold Spring Harb Perspect Biol. 2009;1(6). doi: 10.1101/cshperspect.a001503 20457561 PMC2882119

[5] Canty L, Zarour E, Kashkooli L, François P, Fagotto F. Sorting at embryonic boundaries requires high heterotypic interfacial tension. Nat Commun. 2017;8(1):1–15. doi: 10.1038/s41467-017-00146-x 28761157 PMC5537356

[6] Rieu JP, Sawada Y. Hydrodynamics and cell motion during the rounding of two dimensional hydra cell aggregates. Eur Phys J B. 2002;27(1):167–172. doi: 10.1140/epjb/e20020142

[7] Steinberg MS. Differential adhesion in morphogenesis: a modern view. Curr Opin Genet Dev. 2007;17(4):281–286. doi: 10.1016/j.gde.2007.05.002 17624758

[8] Krieg M, Arboleda-Estudillo Y, Puech PH, Käfer J, Graner F, Müller DJ, et al. Tensile forces govern germ-layer organization in zebrafish. Nat Cell Biol. 2008;10(4):429–436. doi: 10.1038/ncb1705 18364700

[9] Mombach, Glazier, Raphael, Zajac. Quantitative comparison between differential adhesion models and cell sorting in the presence and absence of fluctuations. Phys Rev Lett. 1995;75(11):2244–2247. doi: 10.1103/PhysRevLett.75.2244 10059250

[10] Foty RA, Pfleger CM, Forgacs G, Steinberg MS. Surface tensions of embryonic tissues predict their mutual envelopment behavior. Development. 1996;122(5):1611–1620. doi: 10.1242/dev.122.5.1611 8625847

[11] Krens SFG, Heisenberg CP. Cell sorting in development. Curr Top Dev Biol. 2011;95:189–213. doi: 10.1016/B978-0-12-385065-2.00006-2 21501752

[12] Graner F, Glazier JA. Simulation of biological cell sorting using a two-dimensional extended Potts model. Phys Rev Lett. 1992;69(13):2013–2016. doi: 10.1103/PhysRevLett.69.2013 10046374

[13] Glazier JA, Graner F. Simulation of the differential adhesion driven rearrangement of biological cells. Phys Rev E. 1993;47(3):2128–2154. doi: 10.1103/PhysRevE.47.2128 9960234

[14] Steinberg MS. Does differential adhesion govern self-assembly processes in histogenesis? Equilibrium configurations and the emergence of a hierarchy among populations of embryonic cells. J Exp Zool. 1970;173(4):395–433. doi: 10.1002/jez.1401730406 5429514

[15] Méhes E, Vicsek T. Segregation mechanisms of tissue cells: from experimental data to models. Complex Adapt Syst Model. 2013;1(1):1–13.

[16] Belmonte JM, Thomas GL, Brunnet LG, de Almeida RMC, Chaté H. Self-Propelled Particle Model for Cell-Sorting Phenomena. Phys Rev Lett. 2008;100(24):248702. doi: 10.1103/PhysRevLett.100.248702 18643634

[17] Nakajima A, Ishihara S. Kinetics of the cellular Potts model revisited. New J Phys. 2011;13(3):033035. doi: 10.1088/1367-2630/13/3/033035

[18] Beatrici CP, Brunnet LG. Cell sorting based on motility differences. Phys Rev E. 2011;84(3):031927. doi: 10.1103/PhysRevE.84.031927 22060423

[19] Voss-Böhme A, Deutsch A. The cellular basis of cell sorting kinetics. J Theor Biol. 2010;263(4):419–436. doi: 10.1016/j.jtbi.2009.12.011 20026134

[20] Méhes E, Mones E, Németh V, Vicsek T. Collective Motion of Cells Mediates Segregation and Pattern Formation in Co-Cultures. PLoS ONE. 2012;7(2):e31711. doi: 10.1371/journal.pone.0031711 22359617 PMC3280994

[21] Harris AK. Is Cell sorting caused by differences in the work of intercellular adhesion? A critique of the Steinberg hypothesis. J Theor Biol. 1976;61(2):267–285. doi: 10.1016/0022-5193(76)90019-9 985668

[22] Steinberg MS. Reconstruction of tissues by dissociated cells. Some morphogenetic tissue movements and the sorting out of embryonic cells may have a common explanation. Science. 1963;141(3579):401–408. doi: 10.1126/science.141.3579.401 13983728

[23] Lamorgese AG, Mauri R. Diffuse-interface modeling of phase segregation in liquid mixtures. Int J Multiph Flow. 2008;34(10):987–995. doi: 10.1016/j.ijmultiphaseflow.2008.03.003

[24] Voorhees PW. The theory of Ostwald ripening. J Stat Phys. 1985;38(1):231–252. doi: 10.1007/BF01017860

[25] Hardy SC, Voorhees PW. Ostwald ripening in a system with a high volume fraction of coarsening phase. Metall Trans A. 1988;19(11):2713–2721. doi: 10.1007/BF02645806

[26] Naso A, Náraigh L. A flow-pattern map for phase separation using the Navier-Stokes Cahn-Hilliard model. Eur J Mech. 2017;72.

[27] Witkowski T, Backofen R, Voigt A. The influence of membrane bound proteins on phase separation and coarsening in cell membranes. Phys Chem Chem Phys. 2012;14(42):14509–14515. doi: 10.1039/c2cp41274h 22801988

[28] Garcke H, Niethammer B, Rumpf M. Transient Coarsening Behaviour In The Cahn-Hilliard Model. Acta Mater. 2003;51. doi: 10.1016/S1359-6454(03)00087-9

[29] Zhang Y, Thomas GL, Swat M, Shirinifard A, Glazier JA. Computer Simulations of Cell Sorting Due to Differential Adhesion. PLoS ONE. 2011;6(10):e24999. doi: 10.1371/journal.pone.0024999 22028771 PMC3196507

[30] Osborne JM, Fletcher AG, Pitt-Francis JM, Maini PK, Gavaghan DJ. Comparing individual-based approaches to modelling the self-organization of multicellular tissues. PLoS Comput Biol. 2017;13(2):e1005387. doi: 10.1371/journal.pcbi.1005387 28192427 PMC5330541

[31] Strandkvist C, Juul J, Baum B, Kabla AJ, Duke T. A kinetic mechanism for cell sorting based on local variations in cell motility. Interface Focus. 2014;4(6). doi: 10.1098/rsfs.2014.0013 25485079 PMC4213444

[32] Durand M. Large-scale simulations of biological cell sorting driven by differential adhesion follow diffusion-limited domain coalescence regime. PLoS Comput Biol. 2021;17(8):e1008576. doi: 10.1371/journal.pcbi.1008576 34398883 PMC8389523

[33] Kabla AJ. Collective cell migration: leadership, invasion and segregation. J R Soc Interface. 2012;9(77):3268–3278. doi: 10.1098/rsif.2012.0448 22832363 PMC3481577

[34] Beatrici CP, de Almeida RMC, Brunnet LG. Mean-cluster approach indicates cell sorting time scales are determined by collective dynamics. Phys Rev E. 2017;95(3):032402. doi: 10.1103/PhysRevE.95.032402 28415271

[35] Krajnc M. Solid–fluid transition and cell sorting in epithelia with junctional tension fluctuations. Soft Matter. 2020;16(13):3209–3215. doi: 10.1039/C9SM02310K 32159536

[36] Schötz EM, Burdine RD, Jülicher F, Steinberg MS, Heisenberg CP, Foty RA. Quantitative differences in tissue surface tension influence zebrafish germ layer positioning. HFSP J. 2008;2(1):42–56. doi: 10.2976/1.2834817 19404452 PMC2640996

[37] Beysens DA, Forgacs G, Glazier JA. Cell sorting is analogous to phase ordering in fluids. Proc Natl Acad Sci USA. 2000;97(17):9467–9471. doi: 10.1073/pnas.97.17.9467 10944216 PMC16887

[38] Cochet-Escartin O, Locke TT, Shi WH, Steele RE, Collins EMS. Physical Mechanisms Driving Cell Sorting in Hydra. Biophys J. 2017;113(12):2827–2841. doi: 10.1016/j.bpj.2017.10.045 29262375 PMC5771031

[39] Vishwakarma M, Spatz JP, Das T. Mechanobiology of leader–follower dynamics in epithelial cell migration. Curr Opin Cell Biol. 2020;66:97–103. doi: 10.1016/j.ceb.2020.05.007 32663734

[40] Fujimori T, Nakajima A, Shimada N, Sawai S. Tissue self-organization based on collective cell migration by contact activation of locomotion and chemotaxis. Proc Natl Acad Sci USA. 2019;116(10):4291–4296. doi: 10.1073/pnas.1815063116 30782791 PMC6410881

[41] Berry J, Brangwynne CP, Haataja M. Physical principles of intracellular organization via active and passive phase transitions. Reports on Progress in Physics. 2018-02;81(4):046601. doi: 10.1088/1361-6633/aaa61e 29313527

[42] Rossbach P, Böhme HJ, Lange S, Voss-Böhme A. Model-Based Prediction of an Effective Adhesion Parameter Guiding Multi-Type Cell Segregation. Entropy. 2021;23(11):1378. doi: 10.3390/e23111378 34828077 PMC8624153

[43] Fijan N, Sulimanović D, Bearzotti M, Muzinić D, Zwillenberg LO, Chilmonczyk S, et al. Some properties of the Epithelioma papulosum cyprini (EPC) cell line from carp cyprinus carpio. Annales de Virologie. 1983;134(2):207–220.

[44] Boelsma E, Verhoeven MCH, Ponec M. Reconstruction of a Human Skin Equivalent Using a Spontaneously Transformed Keratinocyte Cell Line (HaCaT). J Invest Dermatol. 1999;112(4):489–498. doi: 10.1046/j.1523-1747.1999.00545.x 10201534

[45] Meakin P. Diffusion-limited droplet coalescence. Physica A. 1990;165(1):1–18.

[46] Kolb M. Unified Description of Static and Dynamic Scaling for Kinetic Cluster Formation. Phys Rev Lett. 1984;53(17):1653–1656. doi: 10.1103/PhysRevLett.53.1653

