# Supplementary material for: Is cell segregation like oil and water: Asymptotic versus transitory regime
Source: PLoS Comput Biol. 2022 Sep 19;18(9):e1010460. doi: 10.1371/journal.pcbi.1010460 (PMC9484667; doi:10.1371/journal.pcbi.1010460)
Supplement: S1 Text — The Supporting Information S1 Text provides details on the cellular automaton implementation, the Cahn-Hilliard model and its mapping to the cellular automaton, as well as the background of the used 2D-image analysis methods. In addition it contains supporting figures to strengthen our findings. (PDF) [file pcbi.1010460.s001.pdf]

Supporting Information: Is cell segregation like oil and water: asymptotic versus transitory regime

Florian Franke[1,2*], Sebastian Aland[3,2], Hans-Joachim Böhme[1,2], Anja Voss-Böhme[1,2], Steffen Lange[1,2]

1 DataMedAssist, HTW Dresden, Dresden, Germany
2 Faculty of Informatics/Mathematics, HTW Dresden - University of Applied Sciences, Dresden, Germany
3 Faculty of Mathematics and Computer Science, TU Freiberg, Freiberg, Germany

* florian.franke@htw-dresden.de

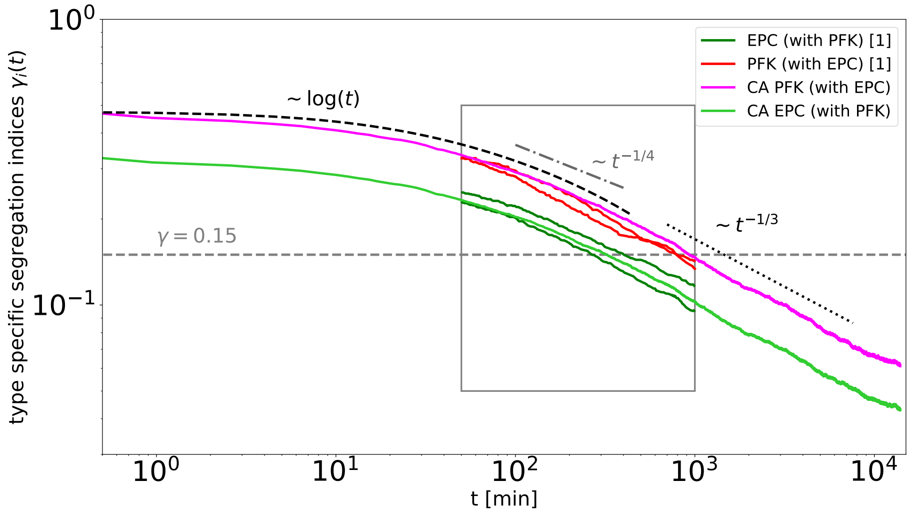

Fig A. The data of Méhes et al. [1] fall into the transitory regime of the cellular automaton. This is shown exemplary for the segregation of PFK with EPC. The simulation with the same parameters as in Fig 1, with time scale of migration $\tau_{\text{PFK-EPC}} \approx 4.2$ min, cell type ratio $N_{\text{PFK}}/N_{\text{EPC}} = 41.2/58.8$, adhesion parameters $(\beta_{\text{PFK-PFK}}, \beta_{\text{EPC-PFK}}, \beta_{\text{EPC-EPC}}) = (-8.06, -6.56, -0.06)$, and 140^2 cells is run for an additional order of magnitude in time such that the segregation indices drop down to $\gamma \approx 0.05$, which is much smaller than the smallest indices $\gamma \approx 0.1$ in Fig 1. Within the given time interval of the experiments (gray box), the cellular automaton shows ambiguous scaling behaviors. The segregation decays logarithmically at the beginning ($\gamma_i \sim 0.58 \log(t)$, black dashed line), followed by an algebraic decay with exponent of 1/4 (gray dash-dotted line) in the transitory regime, and finally by a algebraic decay with exponent 1/3 (black dotted line) at smaller segregation indices $\gamma_{\text{EPC}} < 0.15$, which could be considered as the asymptotic regime.

Cahn-Hilliard Navier-Stokes

The Cahn-Hilliard Navier-Stokes model accurately describes the evolution of two immiscible fluids under flow and diffusion [2, 3]. The model is also used as a typical

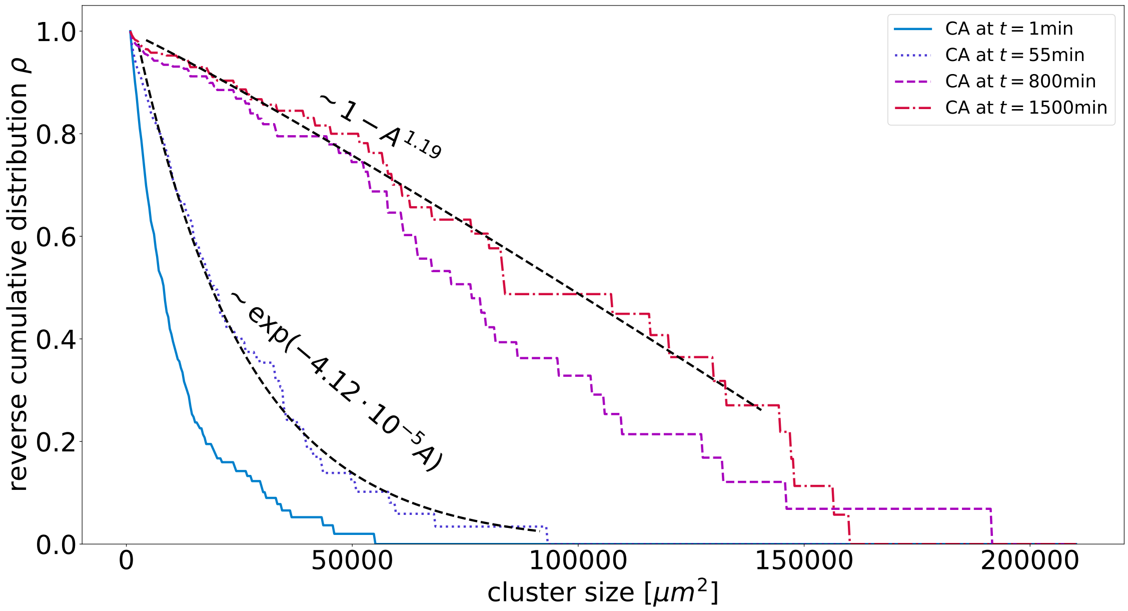

Fig B. The cluster size distribution ρ in the cellular automaton displays different characteristics for the early and the later regime. The reverse cumulative cluster size distributions are shown at four points of segregation. Note that for the early regime, $t = 1$min and $t = 55$min, the reverse cumulative cluster size distribution ρ follows an exponential decay $\exp(-k \cdot A)$. For the later regime, $t = 800$min and $t = 1500$min, the cluster size distribution displays an algebraic decay with exponent ≈ 1.

choice for simulating fluid segregation [4–7]. This model is known for producing an algebraic scaling with the exponent of 1/3 for segregation processes with small length scale [8, 9]. For larger length scales, an exponent of 2/3 can be observed [9]. The complete model is given by the following set of differential equations:

$$\begin{aligned} &\partial_t \Phi + \mathbf{u} \cdot \nabla \Phi = D \Delta \mu, \\ &\mu = -\epsilon^2 \Delta \Phi + \Phi^3 - \Phi, \\ &\rho(\partial_t \mathbf{u} + (\mathbf{u} \cdot \nabla)\mathbf{u}) + \nabla p = \nabla \cdot (\eta(\nabla \mathbf{u} + \nabla \mathbf{u}^T)) + \tilde{\sigma}\epsilon^{-1}\mu\nabla\Phi, \\ &\nabla \cdot \mathbf{u} = 0. \end{aligned} \tag{S1}$$

At small length scales, predefined by the size of the biological cells, the fourth order diffusion in the Cahn-Hilliard equations dominates, such that the influence of flow can be neglected and Eq. (S1) simplifies to:

$$\begin{aligned} &\partial_t \Phi = D \Delta \mu, \\ &\mu = -\epsilon^2 \Delta \Phi + \Phi^3 - \Phi. \end{aligned} \tag{S2}$$

These equations are defined for a domain $\Omega = [0, L_x] \times [0, L_y]$ where L_x and L_y denotes the maximal size of the domain. We define a phase $\Phi : \Omega \to [-1, 1]$ on this domain, where $\Phi \approx 1$ denotes the first fluid, like water, and $\Phi \approx -1$ denotes the second fluid, like oil. Values of $|\Phi| < \Phi_0$ are defined as interface area, e.g. $\Phi_0 = 0.9$. The width of the interface area is proportional to the parameter ϵ:

$$\delta = \operatorname{arctanh}(\Phi_0)\sqrt{2}\epsilon, \tag{S3}$$

see Fig C in S1 Text below.

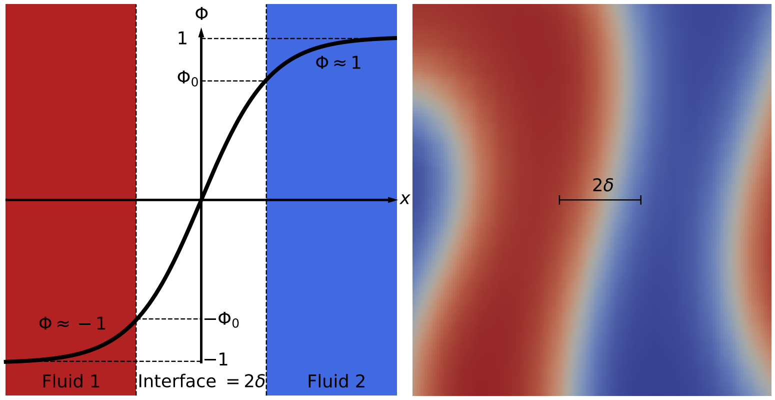

Fig C. Schematic representation of the relationship between Φ and δ and their correlation to the interface area.

The parameter D is the mobility constant and influences the time scale of the diffusion process, which is set by $\tau \sim \frac{\epsilon^2}{D}$. With the phase Φ and the parameter ϵ the chemical potential $\mu(\Phi, \epsilon)$ can be calculated. Each fluid has its own typical parameters like viscosity $\eta_{\Phi=-1}$ and $\eta_{\Phi=_1}$ and density $\rho_{\Phi=-1}$ and $\rho_{\Phi=_1}$. By linear interpolation of the viscosity and density pairs, the functions $\eta(\Phi)$ and $\rho(\Phi)$ can be calculated. Dependent on the types of fluids there is a surface tension σ, which enters the Navier-Stokes equations after a rescaling as $\tilde{\sigma} := \frac{3}{2\sqrt{2}}$ [2, 3].

The implementation of this model follows a special pressure projection scheme with incomplete pressure iterations and an explicit Euler approach described in the paper of Adam et al. [10].

The interface length I for this model is approximated by the Cahn-Hilliard surface energy,

$$I \approx \frac{3}{2\sqrt{2}} \int_{\Omega} \frac{1}{\epsilon} W(\Phi) + \frac{\epsilon}{2} |\nabla \Phi^2|, \tag{S4}$$

$$W(\Phi) = \frac{1}{4}(1 - \Phi^2)^2. \tag{S5}$$

At the start of a simulation, the phase Φ is set randomly in between 0 and 1 following a uniform distribution for each grid point. Then an initial settling process takes place, where no clear phases are observed, since all values of $|\Phi|$ are significantly smaller than 1. In this time span, the measured interface length with Eq. (S4) is irrelevant for our purpose, since there are no clear phases which segregate. This effect is displayed in Panels A-C in the Fig E in S1 Text. If Eq. (S4) yields a plateau over some time, the settling process ceases, two well-mixed phases have established, and the CH behavior can be interpreted as segregation process. Note, that one can also observe a slight local increase of the quantity I in Eq. (S4) instead of a plateau.

Mapping of the cellular automaton model and the Cahn-Hilliard model

We developed a mapping process, to equally start a simulation of the cellular automaton model and the Cahn-Hilliard model and to compare their segregation behaviors. Therefore a match of the time or length scale between both models is needed. Since the cellular automaton initially always segregates logarithmically and the Cahn-Hilliard model algebraically, the time scale can never be the same. However, the length scale can be matched. The length scale in the Cahn-Hilliard model is set by the parameter for the width of the interface area ϵ. Since the interface area in the cellular automaton is sharp, we define an equally wide transition to be from the middle of one cell $\Phi \approx -0.9$ to the middle of a neighboring heterotypic cell $\Phi \approx 0.9$. The length of a side $\triangle x$ of one cell in the cellular automaton can be calculated by the square root of the average of the specific cell areas $A = (A_0 + A_1)/2 \approx 350\mu\text{m}^2$. The transition area in the cellular automaton refers to 2δ, see Eq. (S3), and is equal to one cell side length $\triangle x \approx \sqrt{350}\mu\text{m}$, see Fig D in S1 Text.

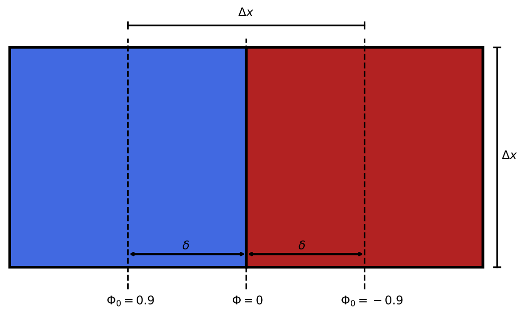

Fig D. Visualization of the mapping of the transition area between the cellular automaton model and the Cahn-Hilliard model. Each square represents a cell in the cellular automaton, where red denotes $i = 0$ and blue $i = 1$ for $i \in W$. The length of one side is defined as $\triangle x$ and can be calculated by the square root of the specific cell type area A_0, A_1. We define the middle of each cell to be equally to each phase $\Phi_0 = 0.9, -0.9$ of the Cahn-Hilliard model. Therefore can the absolute width of the transition area δ be calculated and ϵ can be determined.

Further, the cell type ratio of the cellular automaton can be directly integrated into the Cahn-Hilliard model, by initializing the simulation with an equal phase ratio. The domain size $L_x = L_y$ can be obtained by the number of cells per dimension multiplied with the average size of a cell $N\delta = N\sqrt{350}\mu\text{m}$. Only the mobility constant D needs to be fitted. Since the interface width ϵ is set, the time scales are only influenced by D. Therefore, if D is doubled, the time scale is halved. If the length scales are matched, the initial interface length I will be equal in both models, if both start from a random field. The comparison of both models can be seen in Fig E in S1 Text.

Two point correlation method

The two point correlation method is commonly used to measure the average cluster diameter of cells [1, 11]. Our implementation follows

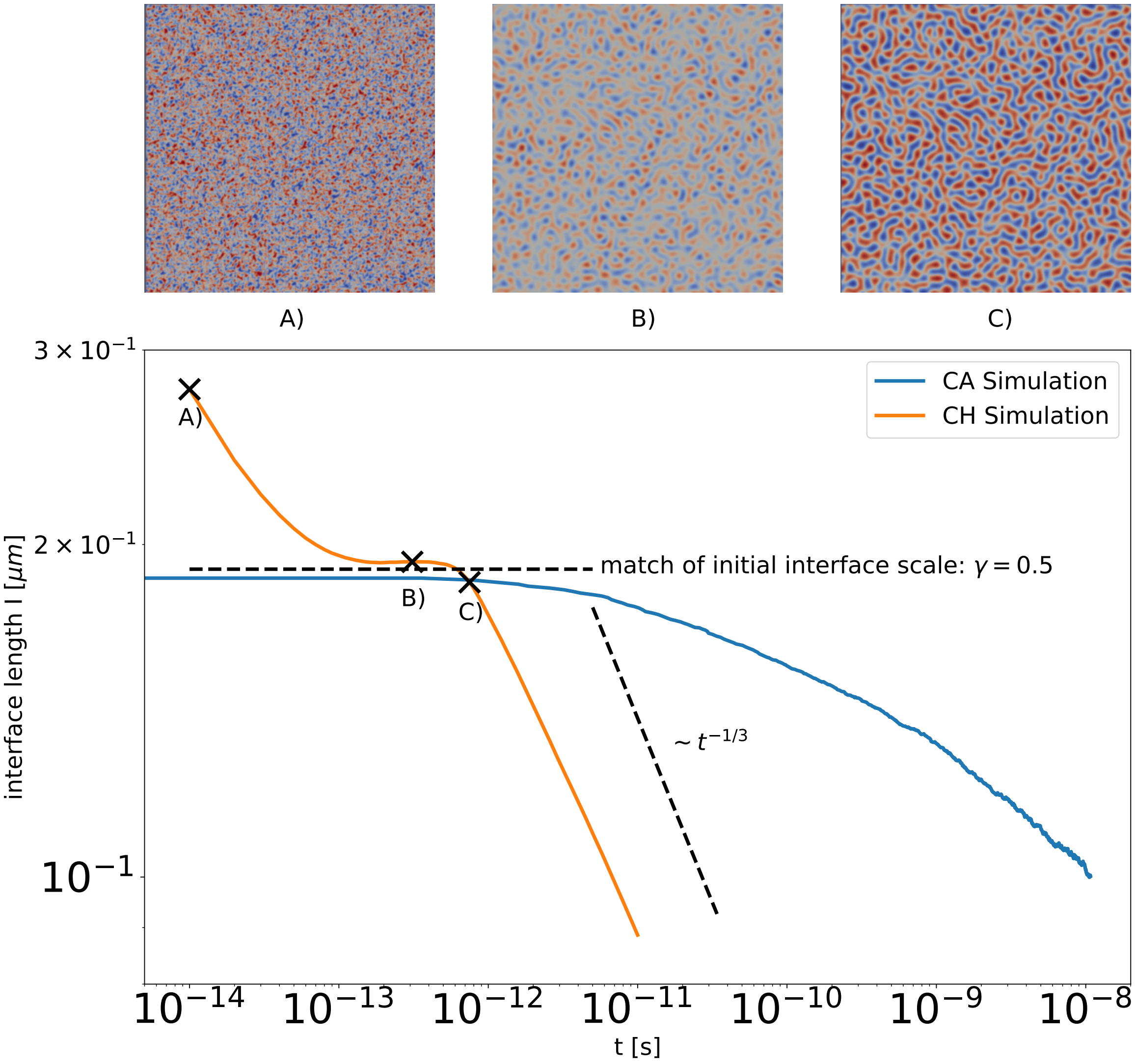

Fig E. The cellular automaton and the Cahn-Hilliard model after an initial settling process start at the same interface length. The simulation of the cellular automaton is started with a random cell mixture, adhesion parameter $\beta = (-1.56, -3.06, -1.56)^T$, time scale of migration $\tau = 1$s, 100^2 cells, and a cell type ratio of 50/50. The Cahn-Hilliard simulation is started from a random, uniformly distributed field with a mobility constant $D = 0.24\mu\text{m}^2/\text{s}$. Note that the Cahn-Hilliard model has always an initial settling process, where the phases get formed, see Panels A and B, concluded by a plateau phase for the interface length where two well-mixed phases can be distinguish, see Panel C. Therefore, the interface length at point C of the Cahn-Hilliards model is matched with the initial interface length in the cellular automaton.

$$C(r,t) = \int_0^{2\pi} C(\mathbf{r},t)d\theta$$
$$C(\mathbf{r},t) = \langle \Phi(\mathbf{r_0},t)\Phi(\mathbf{r_0}+\mathbf{r},t)\rangle_{\mathbf{r_0}} - \langle \Phi(\mathbf{r_0},t)\rangle^2_{\mathbf{r_0}} \qquad \text{(S6)}$$

where the phase $\Phi(\mathbf{r},t)$ is 1 if the first cell type is present at $\mathbf{r}$ and -1 if the other is

present, and $\langle . \rangle_{\mathbf{r_0}}$ denotes the average over all grid points $\mathbf{r_0}$. The average radius of a cluster is defined by the smallest radius at which the correlation becomes zero $C(\mathbf{r}, t) = 0$. Two examples of the correlation function can be seen in Fig J in S1 Text.

Video analysis

Since the details of the video analysis of Méhes et al. [1] are not fully available, we reanalysed the video S5 documenting the segregation of PFK and EPC, see Fig 3 center panels. Through the depicted scale, we identified that $100\mu m$ corresponds to 37 pixel. Since the average size of PFK and EPC cells equals $\sim 350\mu m^2$, a square with a edge length of $\sqrt{350}\mu m \approx 7$ pixel yields the same area. Further, we divided each frame in boxes of 7 by 7 pixels and assigned a cell type to each box. To assign the cell type to each box, we sum the red (PFK) and green (EPC) color channel of each pixels RGB value for each box. We found, that favoring EPC cells in the interpretation of the images by multiplying the red (PFK) sum value with 77% and classifying the entire box as red cell type (PFK) only if this reduced value is greater than the non-reduced green (EPC) sum value leads to stable cell type ratios over time, see Fig G in S1 Text. In contrast, if both pixel color values were treated equally, then too many boxes would be assigned to red (PFK) as Figs F and G in S1 Text highlight.

We further find, in addition to the 77% rule, that single boxes, which are surrounded by the opposite type, should be removed, to gather a representative grid in regards to the original video. Without the removal of single boxes, we find a biological incorrect grid representation of the original experiment, as, especially in the later stages, single boxes of EPC (green) can be seen in the larger clusters of PFK (red), which is not the case for the video, see Panels B and H in Fig G in S1 Text. It follows, that with the removal of single 7^2 pixels boxes, the minimal cluster size for the experiment equals 2 cells. With these technical adjustments, we are able to reproduce the segregation indices and cell type ratios reported by Méhes et al. [1], see Figs H and G in S1 Text.

Note, that the time in the video is very coarsely labeled, as it is limited to full hours. Since some hours included more frames than others, we decided to linearly interpolate the time for each frame. For this, we used the first frame with 1h labeled and the first frame with 16h labeled, which is the latest full hour time stamp. This approximation together with the limited resolution of the published video and the cutoff from the original microscopy images, this explains the small discrepancies between our data and the segregation indices reported in Méhes et al. [1].

Cluster sizes

We compute the distribution ρ of cluster sizes for the models and the experiment, see Fig 4 and B in S1 Text. Since the cellular automaton and the video analysis of the experiment already yield a grid for each time point with assigned cell types, only the output of the Cahn-Hilliard model needs to be converted. Since the total area of simulation for the cellular automaton and the Cahn-Hilliard model are equal, due to our developed mapping, we project the Cahn-Hilliard grid to the grid used by the cellular automaton. Each set of grid points of a cluster has the same type and is connected to every other grid point in this set through a sequence of von-Neumann neighborhoods within the cluster. After identifying each cluster, we count the number of grid points contained in each cluster and compute the corresponding area.

Pseuodo algorithm for the cellular automaton

In order to simulate the cellular automaton in an effective way, we implemented a version with continuous time by applying the idea of the Gillespie-algorithm to the

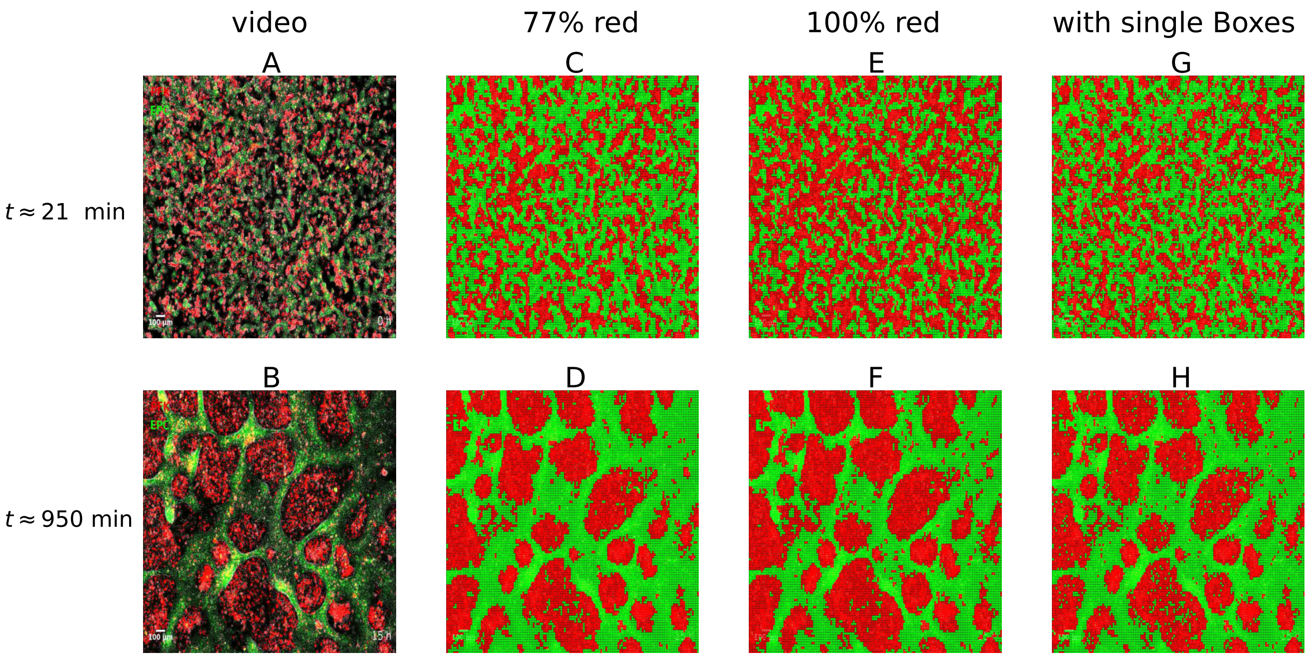

Fig F. Illustration of the color calibration method used in the video analysis. The first row shows a frame from the experiment PFK with EPC at the time $t = 21$ min. The second row shows a frame at the time $t = 950$ min, of the same experiment. A and B display the original frame of the video S5 of Méhes et al. [1]. C and D show the result of the video analysis with the factor 77% for the red channel and when single boxes in opposite-type environment are removed. E and F show the result when the color values per box are treated equally, which corresponds to a factor of 100% for the red channel. G and H show the effect when individual boxes in opposite-type environments are not removed but a factor of 77% for the red channel is used. Comparison of the second and third column justify that the red (PFK) pixel color sum value for each box should be multiplied by 77% and than compared to the green (EPC) pixel color sum value. Comparison of the second and fourth column with the first one justify the removal of single boxes in opposite-type environment.

cellular automaton, instead of using the usual Metropolis algorithm. This improved the performance of our simulations drastically, in comparison to a simple algorithm with discrete time steps and made the calibration to the experiments feasible. The pseudo algorithm used for the cellular automaton reads as follows:

1. Initialise the lattice.
2. Choose random one heterogeneous transition ($\mathbf{x}, \mathbf{y} \in S, |\mathbf{x} - \mathbf{y}| = \Delta x \wedge \xi \neq \xi^{\mathbf{x},\mathbf{y}}$). Transitions with a higher rate $r(\mathbf{x}, \mathbf{y})$, will be chosen with a linear higher probability $P(\xi \rightarrow \xi^{\mathbf{x},\mathbf{y}})$.
3. The two cells of the selected transition will swap there position on the lattice.
4. Δt_{swap} is added to the time.
5. If an end condition is reached, stop here.
6. Else, return to step 2.

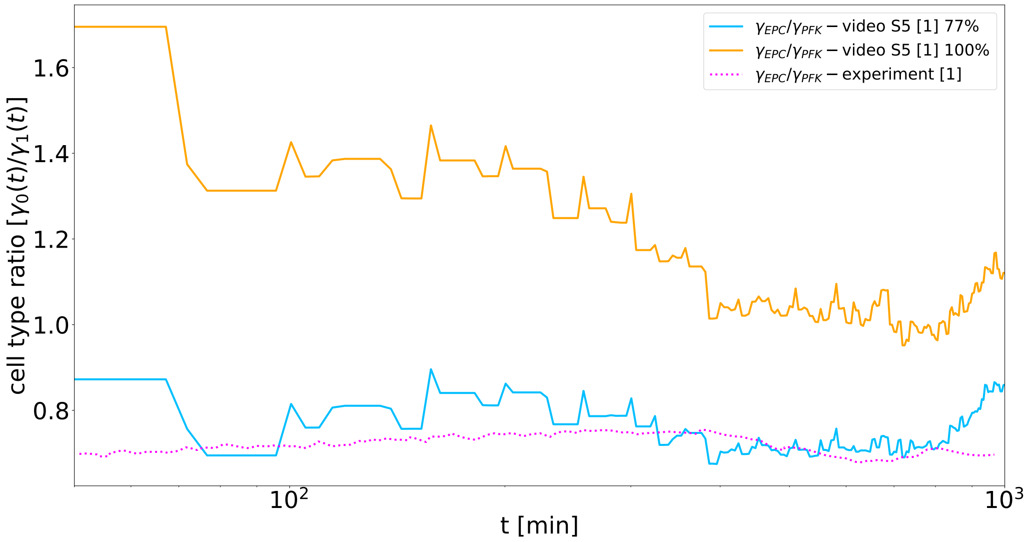

Fig G. Illustration of the color calibration method used in the video analysis. The dotted magenta line shows the cell type ratio reported by Méhes et al. [1]. The solid orange line represents the cell type ratio, if the pixel color sum for red (PFK) and green (EPC) is treated equally. The solid blue line displays the cell type ratio if the red (PFK) pixel color sum value for each box is multiplied by 77% and than compared to the green (EPC) pixel color sum value. If both colors are treated equally (orange line), the cell type ratio varies over time, contradicting the fact that cell numbers in the experiment were kept constant. This supports the use of the 77% factor for the red channel.

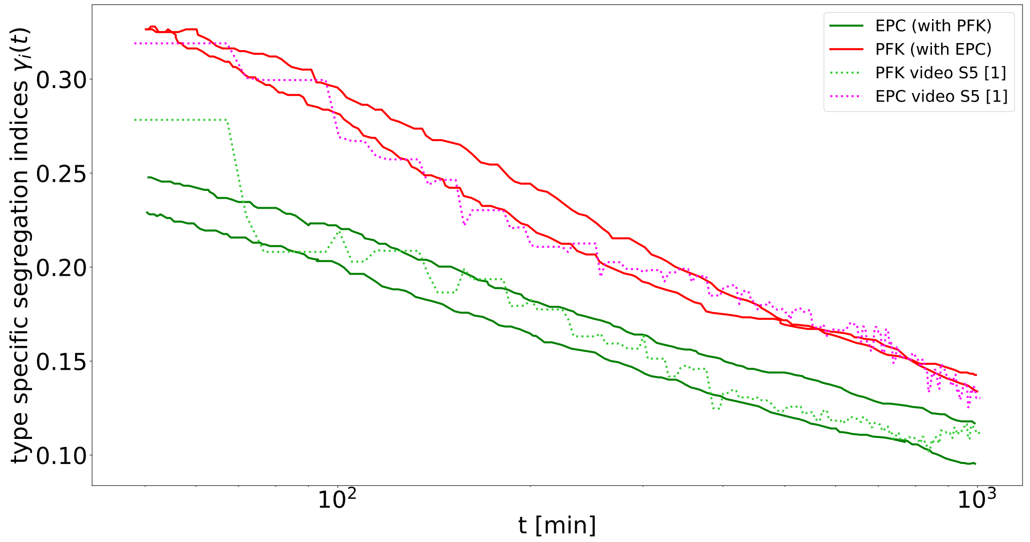

Fig H. The segregation indices computed from our video analysis (dotted lines) fits well the indices reported by Méhes et al. [1] (solid lines) for EPC (green) with PFK (red). The averaged mean squared deviation $\Delta\gamma$ of the result of the video analysis equals 0.0032.

$$P(\mathbf{x},\mathbf{y}) = \frac{r(\mathbf{x},\mathbf{y})}{\sum_B r(e)}, e \in B, r(e) := r(\mathbf{x},\mathbf{y}), \\ B = \{\mathbf{x},\mathbf{y} \in S, |\mathbf{x}-\mathbf{y}| = \Delta x \wedge \xi \neq \xi^{\mathbf{x},\mathbf{y}}\} , \tag{S7}$$

$$\Delta t_{\text{swap}} \sim Exp(\lambda(\xi)), \tag{S8}$$

$$\lambda(\xi) = \sum_{B} \exp(\beta_{\text{sum}}(\mathbf{x}, \mathbf{y}, \xi)). \tag{S9}$$

Here B denotes the set of all possible heterogeneity transitions.

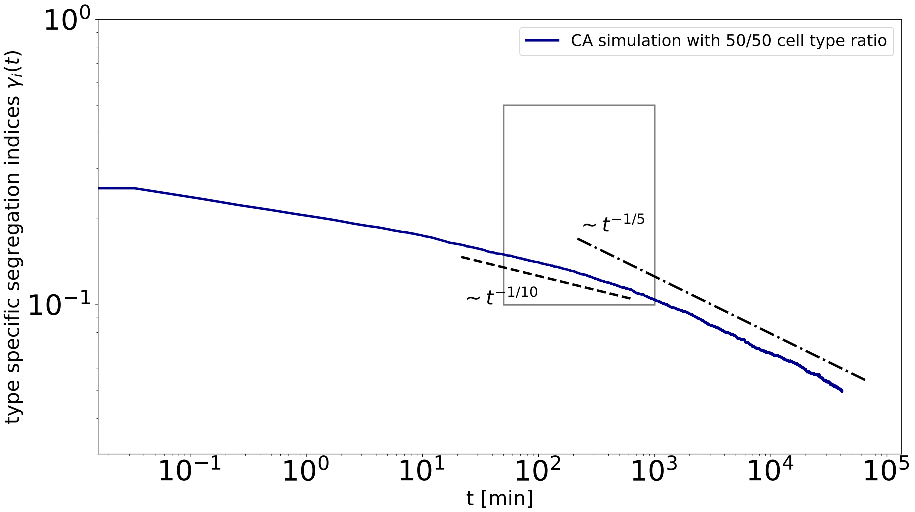

Fig I. Test whether the scaling of the segregation index changes after the observations window. Even the cellular automaton simulation with a very flat initial progression does not reach the asymptotic scaling within the experimental regime, because the scaling still changes after the observation window (gray box) from $t^{-1/10}$ to $t^{-1/5}$. Shown are the segregation indices for a cellular automaton simulation with adhesion parameter $\beta = (0.44, -4.06, 0.44)^T$, time scale of migration $\tau = 1\text{s}$, 140^2 cells, and a cell type ratio of 50/50. The flat early algebraic scaling with exponent 1/10 changes after the observation window of the experiment (gray box) to an algebraic scaling with exponent 1/5. The scaling behavior is thus transitory. Note, that the chosen adhesion parameters translate to effective parameters $db = 0$ and $b^* = 9$ and correspond to the simulation with the lowest pseudo-algebraic scaling exponent in Fig 8.

publication	environment	segregation index algebraic exponent	average cluster diameter algebraic exponent
Naso and Náraigh [9]	Cahn-Hilliard	1/3 (asymptotic)<br>$\geq 1/6$ (transitory)	
Naso and Náraigh [9]<br>Witkowski et al. [4]<br>Garcke et al. [8]	Cahn-Hilliard<br>Navier-Stokes	2/3 (asymptotic)<br>$\geq 1/6$ (transitory)	
Glazier and Graner [12, 13]	CPM	logarithmic	
Nakajima and Ishihara [11]	CPM	1/3 (even mixtures)<br>1/4 (uneven mixtures)	1/3 (even mixtures)<br>1/4 (uneven mixtures)
Durand [14]	CPM	1/4	
Cochet-Escartin et al. [15]	3D CPM	0.5	
Beatrici and Brunnet [16]	boids model	0.18 to 0.22 or logarithmic	
Strandkvist et al. [17]	boids model	0.025 to 0.17	
Belmonte et al. [18]	self-propelled particle model with velocity alignment	≤ 0.18	
Beatrici et al. [19]	active particle approach		1/4 (without collective motion)<br>1/2 (collective motion)
Krajnc [20]	vertex model	$\leq 1/4$	
Krieg et al [21]	experiment		1/10
Cochet-Escartin et al. [15]	3D experiment	0.74	
Méhes et al. [1]	experiment	0.31	0.5 to 0.74

Table A. Summary of the scalings previously published of studies used in the introduction.

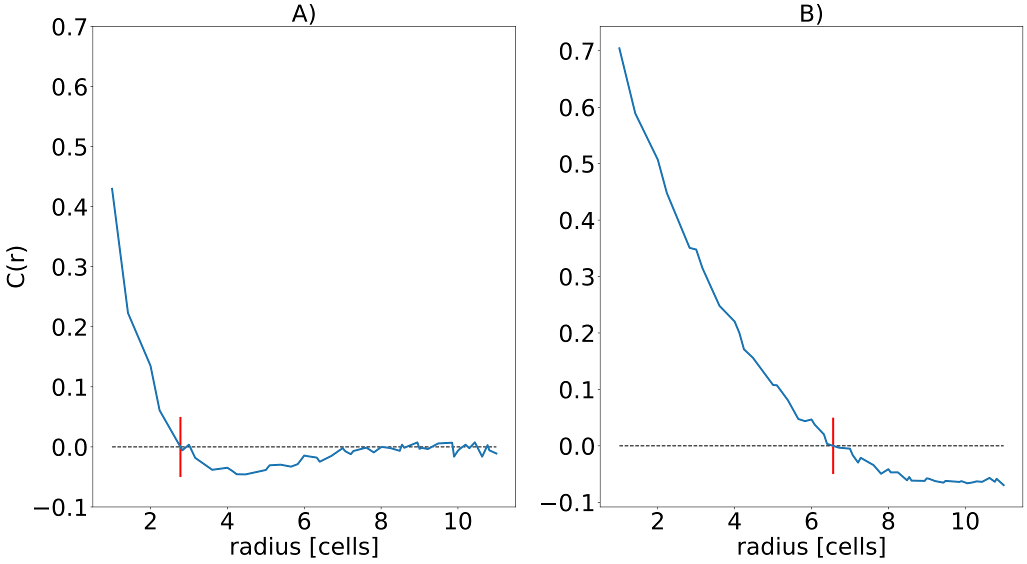

Fig J. Exemplary development of the two point correlation in the cellular automaton. Panel A shows the correlation $C(\mathbf{r})$ at the time $t = 55$min, which is similar to the start of the experiment. Panel B shows the correlation $C(\mathbf{r})$ at the time $t = 800$min, which is similar to the end of the experiment. The radius r is given in cellular automaton cells of width $\Delta x = \sqrt{350}\mu$m. The average radius of a cluster is defined by the smallest radius at which the correlation becomes zero $C(\mathbf{r}) = 0$ (red line).

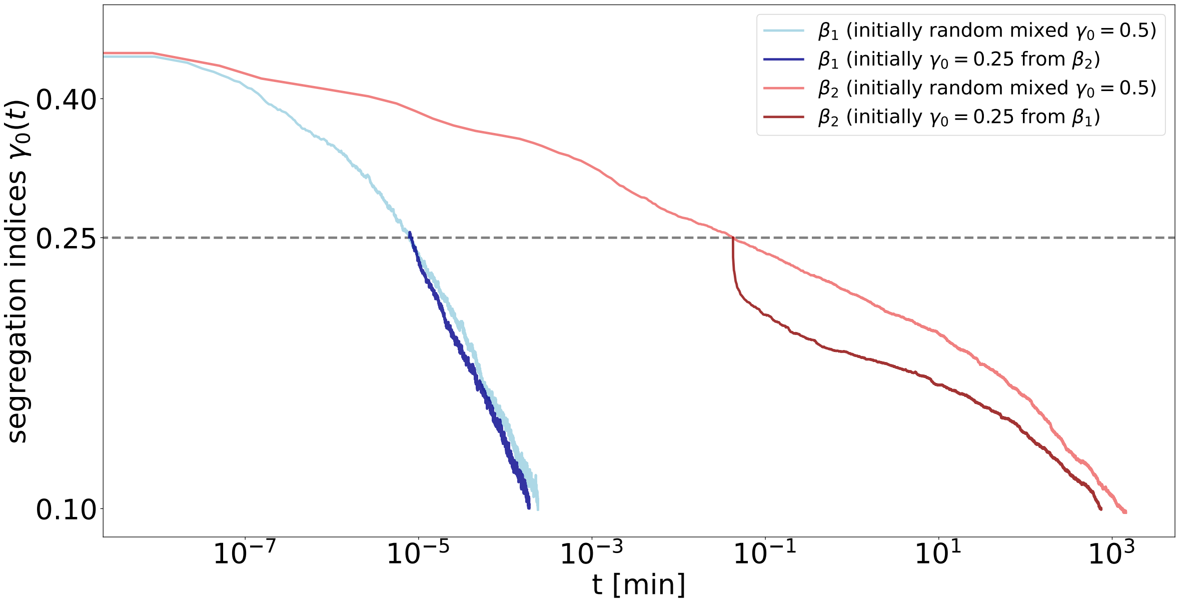

Fig K. Illustration of the influence of initial conditions on the segregation process: Exemplary comparison of the segregation for different initial conditions in the cellular automaton. For each set of adhesion parameters $\boldsymbol{\beta_1} = (0.44, -4.06, 0.44)^T$ (blue lines), $\boldsymbol{\beta_2} = (-8.0, -5.5, 0.0)^T$ (red lines) the simulation is started from a random initial condition $\gamma_0 \approx 0.5$ (brighter colored lines) and from a partially sorted field $\gamma_0 \approx 0.25$ (darker colored lines), which resulted from segregation with the respective other set of adhesion parameters. All simulations used 100^2 cells and a 50/50 ratio (only γ_0 shown). The parameters $\boldsymbol{\beta_1}$ are the γ-ρ-fitted adhesion parameters, see Fig 6, the parameters $\boldsymbol{\beta_2}$ are from Fig I in S1 Text.

References

The numbering of the references used in the Supporting Information S1 Text refers to the References list in the main text:

References

1. Méhes E, Mones E, Németh V, Vicsek T. Collective Motion of Cells Mediates Segregation and Pattern Formation in Co-Cultures. PLoS ONE. 2012;7(2):e31711. doi:10.1371/journal.pone.0031711.

2. Aland S, Boden S, Hahn A, Klingbeil F, Weismann M, Weller S. Quantitative comparison of Taylor flow simulations based on sharp-interface and diffuse-interface models. Int J Numer Methods Fluids. 2013;73. doi:10.1002/fld.3802.

3. Aland S, Voigt A. Benchmark computations of diffuse interface models for two-dimensional bubble dynamics. Int J Numer Methods Fluids. 2012;69(3):747–761. doi:10.1002/fld.2611.

4. Witkowski T, Backofen R, Voigt A. The influence of membrane bound proteins on phase separation and coarsening in cell membranes. Phys Chem Chem Phys. 2012;14(42):14509–14515. doi:10.1039/C2CP41274H.

5. Hardy SC, Voorhees PW. Ostwald ripening in a system with a high volume fraction of coarsening phase. Metall Trans A. 1988;19(11):2713–2721. doi:10.1007/BF02645806.

6. Voorhees PW. The theory of Ostwald ripening. J Stat Phys. 1985;38(1):231–252. doi:10.1007/BF01017860.

7. Rogers TM, Desai RC. Numerical study of late-stage coarsening for off-critical quenches in the Cahn-Hilliard equation of phase separation. Phys Rev B. 1989;39(16):11956–11964. doi:10.1103/PhysRevB.39.11956.

8. Garcke H, Niethammer B, Rumpf M. Transient Coarsening Behaviour In The Cahn-Hilliard Model. Acta Mater. 2003;51. doi:10.1016/S1359-6454(03)00087-9.

9. Naso A, Náraigh L. A flow-pattern map for phase separation using the Navier-Stokes Cahn-Hilliard model. Eur J Mech. 2017;72. doi:10.1016/j.euromechflu.2018.08.002.

10. Adam N, Franke F, Aland S. A Simple Parallel Solution Method for the Navier–Stokes Cahn–Hilliard Equations. J Math. 2020;8(8). doi:10.3390/math8081224.

11. Nakajima A, Ishihara S. Kinetics of the cellular Potts model revisited. New J Phys. 2011;13(3):033035. doi:10.1088/1367-2630/13/3/033035.

12. Graner F, Glazier JA. Simulation of biological cell sorting using a two-dimensional extended Potts model. Phys Rev Lett. 1992;69(13):2013–2016. doi:10.1103/PhysRevLett.69.2013.

13. Glazier JA, Graner F. Simulation of the differential adhesion driven rearrangement of biological cells. Phys Rev E. 1993;47(3):2128–2154. doi:10.1103/PhysRevE.47.2128.

14. Durand M. Large-scale simulations of biological cell sorting driven by differential adhesion follow diffusion-limited domain coalescence regime. PLoS Comput Biol. 2021;17(8):e1008576. doi:10.1371/journal.pcbi.1008576.

15. Cochet-Escartin O, Locke TT, Shi WH, Steele RE, Collins EMS. Physical Mechanisms Driving Cell Sorting in Hydra. Biophys J. 2017;113(12):2827–2841. doi:10.1016/j.bpj.2017.10.045.

16. Beatrici CP, Brunnet LG. Cell sorting based on motility differences. Phys Rev E. 2011;84(3):031927. doi:10.1103/PhysRevE.84.031927.

17. Strandkvist C, Juul J, Baum B, Kabla AJ, Duke T. A kinetic mechanism for cell sorting based on local variations in cell motility. Interface Focus. 2014;4(6). doi:10.1098/rsfs.2014.0013.

18. Belmonte JM, Thomas GL, Brunnet LG, de Almeida RMC, Chaté H. Self-Propelled Particle Model for Cell-Sorting Phenomena. Phys Rev Lett. 2008;100(24):248702. doi:10.1103/PhysRevLett.100.248702.

19. Beatrici CP, de Almeida RMC, Brunnet LG. Mean-cluster approach indicates cell sorting time scales are determined by collective dynamics. Phys Rev E. 2017;95(3):032402. doi:10.1103/PhysRevE.95.032402.

20. Krajnc M. Solid–fluid transition and cell sorting in epithelia with junctional tension fluctuations. Soft Matter. 2020;16(13):3209–3215. doi:10.1039/C9SM02310K.

21. Krieg M, Arboleda-Estudillo Y, Puech PH, Käfer J, Graner F, Müller DJ, et al. Tensile forces govern germ-layer organization in zebrafish. Nat Cell Biol. 2008;10(4):429–436. doi:10.1038/ncb1705.
