# Supplementary figures and images for: Is cell segregation like oil and water: Asymptotic versus transitory regime
Source: PLoS Comput Biol. 2022 Sep 19;18(9):e1010460. doi: 10.1371/journal.pcbi.1010460 (PMC9484667; doi:10.1371/journal.pcbi.1010460)

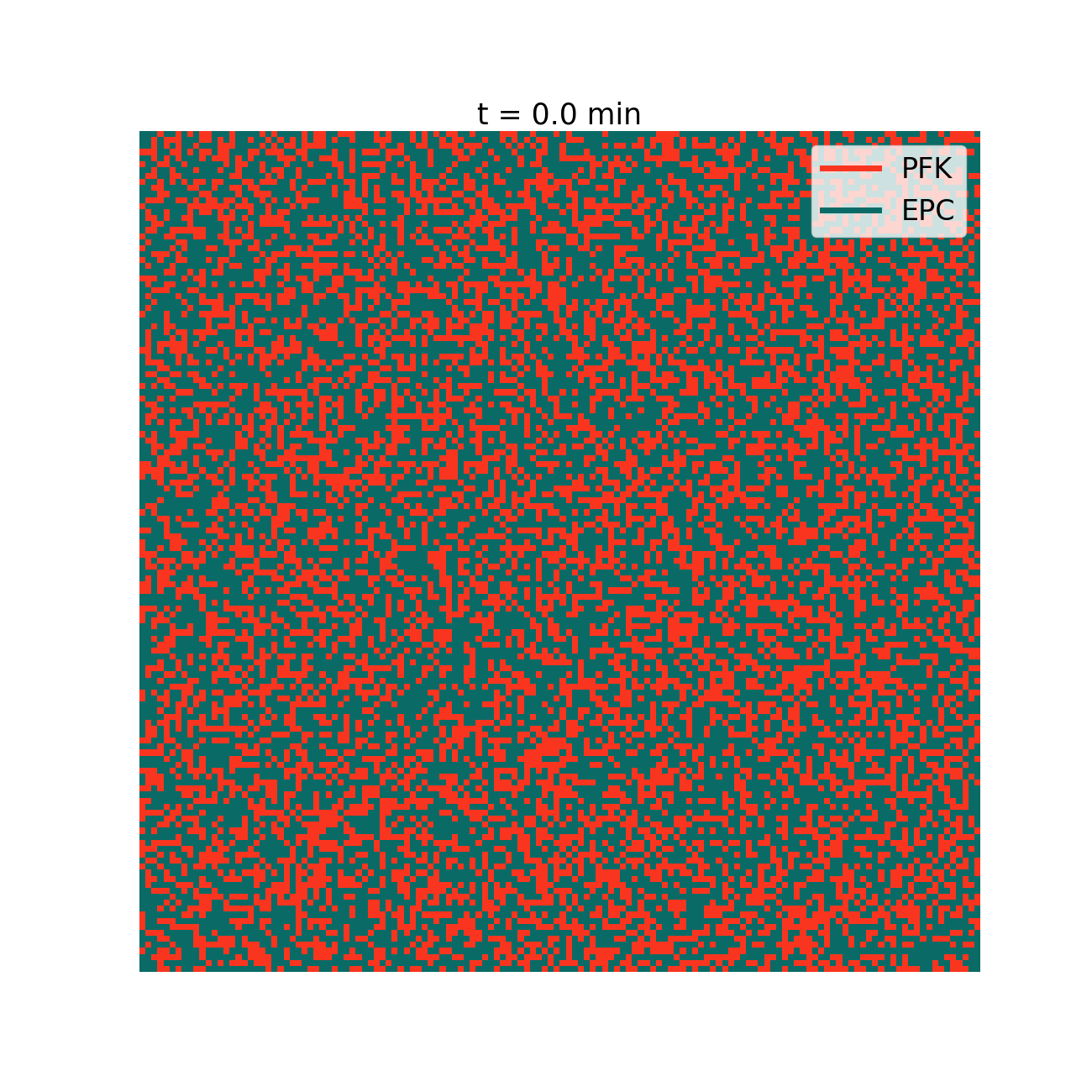

Supplement: S1 Movie — (GIF) [file pcbi.1010460.s002.gif]
